# The potential impact and cost-effectiveness of tobacco reduction strategies for tuberculosis prevention in Canadian Inuit communities

**DOI:** 10.1186/s12916-019-1261-5

**Published:** 2019-02-04

**Authors:** Dieynaba S. N’Diaye, Ntwali Placide Nsengiyumva, Aashna Uppal, Olivia Oxlade, Gonzalo G. Alvarez, Kevin Schwartzman

**Affiliations:** 1 Montreal Chest Institute, Montreal, Quebec Canada; 2 Respiratory Epidemiology and Clinical Research Unit, Centre for Outcomes Research and Evaluation, Research Institute of the McGill University Health Centre, 1001 boulevard Décarie, Room D05.2511, Montreal, Quebec H4A 3J1 Canada; 3 McGill International Tuberculosis Centre, Montreal, Quebec Canada; 4 The Ottawa Hospital Research Institute, Ottawa, Ontario Canada; 5 Department of Medicine, Division of Respirology, The Ottawa Hospital and University of Ottawa, Ottawa, Ontario Canada

**Keywords:** Cost-effectiveness, Decision analysis, Tuberculosis, Tobacco, Inuit

## Abstract

**Background:**

Tuberculosis (TB) remains a significant public health problem in Canadian Inuit communities. In 2016, Canadian Inuit had an incidence rate 35 times the Canadian average. Tobacco use is an important risk factor for TB, and over 60% of Inuit adults smoke. We aimed to estimate changes in TB-related outcomes and costs from reducing tobacco use in Inuit communities.

**Methods:**

Using a transmission model to estimate the initial prevalence of latent TB infection (LTBI), followed by decision analysis modelling, we conducted a cost-effectiveness analysis that compared the current standard of care for management of TB and LTBI without additional tobacco reduction intervention (Status Quo) with (1) increased tobacco taxation, (2) pharmacotherapy and counselling for smoking cessation, (3) pharmacotherapy, counselling plus mass media campaign, and (4) the combination of all these. Projected outcomes included the following: TB cases, TB-related deaths, quality-adjusted life years (QALYs), and health system costs, all over 20 years.

**Results:**

The combined strategy was projected to reduce active TB cases by 6.1% (95% uncertainty range 4.9–7.0%) and TB deaths by 10.4% (9.5–11.4%) over 20 years, relative to the status quo. Increased taxation was the only cost-saving strategy.

**Conclusions:**

Currently available strategies to reduce commercial tobacco use will likely have a modest impact on TB-related outcomes in the medium term, but some may be cost saving.

**Electronic supplementary material:**

The online version of this article (10.1186/s12916-019-1261-5) contains supplementary material, which is available to authorized users.

## Background

Tuberculosis (TB) incidence in Canada has been declining in recent years, to 4.8 cases per 100,000 population in 2016 [1]. Incidence in the Canadian-born non-Indigenous population has declined steadily, but rates remain substantially higher in Indigenous Canadians [1, 2]. A substantial decline in TB incidence was observed in Canadian Inuit communities from the 1960s through the 1980s [2]. However, there has been a resurgence in recent years [2]. For example, in 2016, Canadian Inuit had a TB incidence rate of 170 per 100,000 population [1].

TB programs in Inuit communities face unique environmental, health system, and contextual challenges, including geographic distance, but notably a history of forced separation of persons with TB, and of culturally insensitive health care in the context of colonialism [3]. However, these are not necessarily the only factors contributing to high TB incidence; it is pertinent to consider other potential contributing factors such as tobacco use.

A joint report from the World Health Organization (WHO) and the International Union Against TB and Lung Disease concluded that exposure to tobacco smoke was significantly associated with TB [4]. A modelling study projected that globally, with no improvements in tobacco reduction, smoking would lead to 18 million excess TB cases and 40 million excess TB deaths between 2010 and 2050 [5]. A systematic review identified substantial increases in risks of latent TB infection (LTBI), active TB, and TB-related death associated with smoking [6, 7].

In 2014, Nunavut had the highest proportion of commercial tobacco smokers of all Canadian provinces and territories, with 62% of the population aged over 12 estimated to be current smokers [8]. Among Indigenous adults more specifically, Inuit have the highest smoking prevalence [9]. It is therefore pertinent to consider potential benefits of decreased tobacco use, with respect to TB-related morbidity and mortality. In this modelling study, we examine the potential impact of several different evidence-based approaches to reduce tobacco consumption in Canadian Inuit communities. Strategies considered include (1) increased taxation; (2) pharmacotherapy and counselling; (3) pharmacotherapy, counselling, and mass media; and (4) the combination of all these [10–12]. We predict the impact of these strategies on TB-related health outcomes and attendant costs and cost-effectiveness.

## Methods

### Model overview

#### Dynamic model

Due to the high rates of prior TB exposure in the Inuit population, we first developed and validated a dynamic model that reflects TB epidemiology in Inuit communities in recent decades. Some key pathogenetic values (the contact rate, rapid progression from latent infection to active disease, and protective immunity from previous infection) were also refined and calibrated using this initial dynamic model (Additional file 1: Figure S1), based on observed epidemiologic data from Northern Canada from relevant time periods [13–15].

Four different phases of the TB epidemic were simulated. The first phase involved seeding the model, which corresponds to the historic introduction of the first infectious TB case into a fully susceptible population; this was followed by simulation over several decades to attain equilibrium where TB incidence and smoking prevalence were stable [16]. We used reported observations from this period to estimate the beta parameter (the rate of effective contact between infectious and non-infectious persons per unit of time). We considered that this first phase ended in 1948.

The second phase of the simulation covered the period 1948–1967. In this period, TB incidence declined and smoking prevalence was documented to be stable [16]. Antibiotic use was limited. However, living conditions began to improve, and this was captured by reduction in the beta parameter [17]. In order to calibrate the rate at which the beta declined, we used documented TB incidence and annual risk of infection (ARI) surveys from the Canadian Eastern Arctic [18, 19]. The first two phases marked the period before widespread antibiotic use in Arctic Canada. The third phase covered a period of decline in TB incidence and smoking prevalence from 1967 to 2000, while the fourth phase captured a period of increasing TB incidence from 2000 to 2017 (Additional file 1: Figure S2). It is early in the third phase that current, standard TB-related interventions (i.e., active case finding, smear and culture-based diagnosis, modern drug treatment and INH prophylaxis) were widely adopted. The model was calibrated during this period of declining TB incidence (1967–2000) using observed incidence and ARI [19].

We continued the calibration during 2000–2017, a period that included outbreaks of TB in Nunavut and Nunavik [20, 21]. At the end of the dynamic simulation in 2017, key model outputs such as population distribution in each TB-related health state and other model parameters such as the annual risk of infection were stratified by smoking status. These parameters were then used to populate the Markov decision analysis model used to predict outcomes and costs from 2017 onward.

#### Decision analysis model

We then developed a Markov decision analysis model using TreeAge Pro software (TreeAge Software Inc., Williamstown, MA), to predict future health outcomes and costs after implementation of the tobacco reduction strategies. These strategies had been highlighted by community, public health, and academic informants working in Nunavut.

The cost effectiveness model simulated a hypothetical cohort of Canadian Inuit with median age 20 years, reflecting the age structure of this population [22], starting in 2017 [23, 24]. Health system costs relating to both TB and tobacco interventions and health outcomes including TB incidence, TB-related death, and quality-adjusted life-years (QALYs) were projected over 20 years with either the status quo or each of the different tobacco reduction interventions. A discount rate of 3% was applied to all projected future outcomes. A simplified representation of the decision analysis model is shown in Additional file 1: Figure S3.

### Data inputs for the decision analysis model

#### TB pathogenetic and epidemiologic parameters

Key parameters related to TB pathogenesis, epidemiology, and treatment are shown in Table 1. Additional detail is provided in the Additional file 1.

**Table 1:** Decision analysis model parameters related to the natural history, epidemiology, and treatment of LTBI and TB

Estimates		Reference
Probabilities	Probability (%)*	
•Probability of diagnosing LTBI	25	Assumption
•Probability of completing LTBI treatment among those who start it	70.8	[69]
•Probability of reactivation of longstanding LTBI to TB disease (non-smokers)	0.05/year	[70–72]
•Probability of rapid progression following TB infection (non-smokers)**	4.5/year for 2 years	Dynamic model^#^
•Probability of diagnosing active disease	90	Assumption
•Probability of spontaneous resolution of untreated TB disease	25	[16]
•Probability of relapse following spontaneously resolved TB	2.5	[16, 73]
•Probability of dying from untreated TB	19/year	[74, 75]
•Probability of relapse after treated TB disease in first year	1.5	[76]
•Protective immunity from previous TB disease	55	Dynamic model^#^
QALYs for LTBI and active TB	Value (range)	
•Utility score for individual with active disease (treated)	0.85 (0.70–0.90)	[40]
•Utility score for individual with active disease (untreated)	0.68 (0.65–0.72)	[39]
•Utility score for individual with latent infection, during treatment	0.97 (0.95–1.00)	[40]
•Utility score for individual with latent infection (untreated)	1	Assumption^†^

*INH* isoniazid, *LTBI* latent tuberculosis infection, *QALYs* quality-adjusted life-years

^*^Probabilities are one-time percentages, unless stated otherwise

^**^After 2 years, risk of developing active TB falls to 0.05% annually

^†^Persons with LTBI are asymptomatic

^#^Calibrated in dynamic model

#### Tobacco use as a risk factor for TB

The effects of smoking on the pathogenesis of TB were obtained from two sources: (1) a systematic review which reported a relative risk of acquiring TB infection of 1.9 (95% CI 1.6–2.3) comparing smokers with non-smokers and a relative risk of death from TB of 2.6 (95% CI 1.8–3.6) for smokers compared to non-smokers [6] and (2) a modelling study by the same authors that estimated the relative risk of developing active TB conditional on prior infection (RR = 1.5; 95% CI 1.3–1.7).

#### Tobacco reduction strategies

There are several effective tobacco reduction measures recommended by the WHO Framework Convention on Tobacco Control [25, 26]. Of these, we considered four effective and widely used strategies. Evidence regarding their feasibility, acceptability, and effectiveness in Indigenous communities has also been reviewed by Chamberlain et al. [27] and Minichiello et al. [28]. Details are provided in Table 2 and the Additional file 1. All scenarios were compared to a status quo scenario that included the current standard of care for TB management, described above, as well as the background smoking cessation rate. Historical data from the Canadian Inuit population indicate a steady decline in smoking rates, at an average of 1.2% per year between 1967 and 2000 [8, 29–37]. After 2000, smoking prevalence began to level off and was 62% in 2013 as reported by Statistics Canada [8]. To inform smoking trends after 2000, we used data from Nunavut which showed a continued decrease in smoking, but at a lower average rate of 0.8% annually [38]. We assumed that in the status quo scenario, current approaches including the level of taxation would remain unchanged for the duration of the analysis, as would current smoking cessation rates. The annual rates of decline shown for the various tobacco reduction interventions incorporate this background level of smoking cessation. We assumed that once smokers became non-smokers, they then faced the same TB-related risks as never-smokers.

**Table 2:** Tobacco reduction strategies

Scenario name	Brief description of strategy	Expected reduction in smoking prevalence*	Duration of intervention and its effect	Reference
Pharmacotherapy and counselling	Pharmacotherapy intervention to stop smoking comprised of 12 weeks nicotine replacement therapy (NRT) and varenicline medication. Group and individual counselling was offered to participants to enhance odds of quitting. Content and duration of counselling sessions were obtained from a meta-analysis of smoking cessation interventions with individuals undergoing treatment or recovery and dosage of varenicline and NRT prescriptions were modelled as per Inuit and Canadian standards.	1.5% per year for 3 years	One time opportunity for smokers to join pharmacotherapy and counselling programs. Benefit was assumed to last for 3 years (i.e., smoking prevalence falls at an annual rate of 1.5% for 3 consecutive years). Beyond this period the prevalence declines at the background rate of 0.8% annually.	[46, 77, 78]
Pharmacotherapy, counselling and mass media	Pharmacotherapy and counselling components of this strategy were similar to the above strategy. We used pooled risk ratios for cessation from two randomized trials involving Indigenous people in Australian communities receiving national tobacco campaigns supplemented by other Indigenous specific campaigns such as “Break the chain”.	1.9% per year for 3 years	One time opportunity for smokers to join pharmacotherapy and counselling programs accompanied by a mass media campaign. Benefit was assumed to last for 3 years (i.e., smoking prevalence falls at an annual rate of 1.9% for 3 consecutive years). Beyond this period the prevalence declines at the background rate of 0.8% annually.	[49]
Increased taxation on commercial cigarettes	A legislative approach to commercial tobacco control through increased taxation which has no associated costs from the perspective of the healthcare system. To measure the impact of this policy change vis-à-vis commercial cigarette demand; we used reported price elasticity of demand (i.e., the extent to which cigarette use falls or rises after increases or decreases in price)	7% after 3 years increasing to 14% after 10 years^†^	One time 25% increase in tobacco taxation was assumed to have greater immediate effect in the first 3 years and the effect would then decline over time (i.e., smoking prevalence decreases by 7% over 3 years, and after 10 years it decreases by a total of 14%). Beyond this period the prevalence declines at the background rate of 0.8% annually.	[12, 63, 79, 80]
All strategies (Taxation + mass media + pharmacotherapy + Counselling)	Multifaceted strategy combining all the above strategies: the measure of effect of reduction in smoking prevalence is derived from the effect of increased taxation multiplied by 3-year effect (risk ratio) of pharmacotherapy combined with mass media	5.6% annually for 3 years and 2.6% annually for subsequent 7 years	5.6% annual reduction in smoking prevalence for the first 3 years followed by 2.6% annual reduction up to year 10 after one-time interventions. Beyond this period the prevalence declines at the background rate of 0.8% annually.	[77, 78, 81]

*This table presents annual rates of reduction in smoking prevalence resulting from once off interventions of pharmacotherapy and/or mass media whose effect lasts 3 years

^†^The effect of a once off 25% taxation increase on smoking prevalence is presented at year 3 and year 10, beyond this period, prevalence declines at the background rate of 0.8% annually

#### Health utilities

We used health utility scores based on the time spent with or without active TB or latent TB infection to estimate QALYs. As our model only considered health outcomes related to TB, our QALY estimates included only those related to TB health states and not to other conditions related to smoking. For untreated active TB, we used a utility weight of 0.68 (0.65–0.72) [39]; we used a utility weight of 0.85 (0.70–0.90) for treated active TB, both obtained from Canadian studies [40]. For untreated LTBI, we assumed a utility weight of 1 and used a utility weight of 0.97 for those undergoing LTBI treatment, also based on Canadian data [40].

#### Costs

##### Health system costs for active and latent TB

Where possible, TB-related health system costs were obtained from published sources from Nunavut, Canada. These included costs related to TB diagnosis, hospitalization, treatment (drug costs and administration), and treatment-related adverse events. Diagnostic costs related to active TB disease included chest radiography, sputum analysis, and hospitalization in situations when a person presented with symptoms suggestive of TB. The management of active TB also included the cost of transfers to Ottawa for patients with complications and/or complex medical situations (estimated at 4% of active TB cases) [41, 42]. Diagnostic costs related to LTBI included physical examination, tuberculin skin test (TST), and chest X-ray (CXR) for those who are TST positive [41–43]. Costs were adjusted to 2017 Canadian dollars, with details provided in the Additional file 1 [44]. Selected cost estimates are included in Table 3.

**Table 3:** TB-related health system costs (2017 Canadian $)

	Value	Range	Reference
Health system costs (including material and health care worker time/salary)			
•Tuberculin skin test	$18.22		[82]
•INH (9-month regimen)	$182.01		[83]
•Major adverse reaction to Isoniazid	$14,937		[82]
•Chest X-ray	$68.00		[84]
•Three sputum samples analysis (when results are negative)	$29.34		Dynacare
•Three sputum samples analysis (when results are positive)	$80.79		Dynacare
•Spontaneous sputum production	$3.52	(2.65–4.41)	[41]
•Sputum induction	$96.84		[42]
•Xpert® MTB/RIF test for one individual (1 sample)	$134.40		[41]
•Standard 6 month TB medication regimen^*^	$640.84		[83]

*INH* isoniazid, *NRT* nicotine replacement therapy

*The cost includes vitamin B6

##### Costs related to tobacco reduction interventions

Pharmacotherapy doses and costs were derived from Quebec and Nunavut health schemes, where 12 weeks of nicotine replacement therapy (NRT), including patches and chewing gum, is prescribed through government health plans [45].

A meta-analysis of smoking cessation interventions by Prochaska et al. was used to obtain the number and duration of pharmacotherapy counselling sessions [46]. The mean number of counselling sessions offered was 12, with each session lasting between 5 min and 2 h (mean = 42 min, standard deviation = 33 min). The hourly wage of a nurse in Nunavut [41] was used to calculate the total cost of counselling sessions. Key costs and assumptions associated with tobacco reduction interventions are provided in Table 4.

**Table 4:** Tobacco reduction costs (2017 Canadian $)

Costs associated with specific tobacco reduction scenarios	Component cost	Per person cost of intervention	Reference
1) Increased taxation^†^		$0	
2) Pharmacotherapy and counselling			
a. Unit cost of NRT chewing gum	$0.25		[83]
b. Number of doses of NRT chewing gum (*N*)	1008		[45]
c. Cost of treatment with NRT chewing gum (a*b)	$254.32		
d. Unit cost of a nicotine patch	$2.68		[83]
e. Number of doses of nicotine patch (*N*)	84		[45]
f. Cost of treatment with nicotine patch (d*e)	$225.00		
g. Cost of one varenicline 0.5 mg pill	$1.72		[83]
h. Number of doses of varenicline (*N*)	317		
i. Cost of treatment with varenicline (g*h)	$544.29		
j. Number of counselling cessions	12		[46]
k. Duration of each counselling session	42 min		[46]
l. Hourly Nurse wage	$81.39		[41]
m. Cost for complete counselling sessions (j*k*l)	$683.68		
n. Total cost of Pharmacotherapy and counselling (c + f + i + m) per person	$1707.29		
o. Proportion of smokers who made a quit attempt	14.14%		
Pro-rated mean cost of pharmacotherapy and counselling per smoker (n*o: applied to all smokers in the model cohort)		$241.41	
3) Pharmacotherapy, counselling and mass media			
a. Total cost of pharmacotherapy and counselling	$1707.29		
b. Proportion of smokers who made a quit attempt	19.8%		
c. Recommended per capita expenditure on mass media campaigns	$2.05		[47]
Prorated mean cost of pharmacotherapy and counselling per smoker (a*b + c): added to all smokers		$339.78	
4) Multifaceted strategy—pharmacotherapy, counselling, mass media, and taxation		$339.78	

^†^Cost of taxation is incurred by the individual (hence no health system cost)

Mass media influence smokers depending on the scale of the campaigns. The US Centers for Disease Control and Prevention (CDC) have recommended $1.69 ($1–$3) expenditure per capita for mass-reach health communication interventions to be effective [47]. Based on these recommendations, after accounting for inflation, $2.05 per person was applied to the full population (smokers and non-smokers) for scenarios that included mass media campaigns [44].

### Sensitivity analysis

#### One and two-way analyses

We performed univariate sensitivity analyses, varying key model probabilities and costs. We examined the most influential drivers of (a) TB incidence, (b) TB deaths, (c) QALYs gained, and (d) TB-related health system costs over 20 years. We also considered a scenario where the effect of tobacco-related interventions lasted 5 years, rather than three as we assumed in the primary analysis. According to the 2015 Canadian Tobacco, Alcohol and Drugs Survey (CTADS), 50% of Canadian smokers attempted to quit smoking for at least 24 h [38]; we evaluated the effect of improved availability and use of pharmacotherapy interventions by increasing the proportion of smokers using pharmacotherapy to quit smoking to (a) 50% and (b) 75%.

Probabilistic sensitivity analysis (PSA) was conducted by varying parameter estimates over their distributions (see Additional file 1: Table S2), and running 10,000 simulations to generate 95% uncertainty ranges (UR) for all model outputs.

## Results

### Base case

Compared with the status quo, the strategy combining all interventions was associated with an estimated 6.1% (95% UR 4.9–7.0%) reduction in TB incidence over 20 years: 17.72/1000 persons (14.06/1000–22.32/1000) vs. 18.88/1000 persons (14.81/1000–23.92/1000) for the status quo (Table 5). The same combined strategy was projected to reduce TB deaths by 10.4% (95% UR 9.5–11.4%). Increased taxation was projected to reduce TB incidence by 2.4% (1.9–2.7%) and TB mortality by 3.8% (3.7%–4.4%). A similar pattern was observed in QALYs gained, with an estimated 1 QALY gained per 1000 community members when all interventions were combined. The increased taxation strategy was the only cost saving intervention. Estimated clinical outcomes and costs for all strategies and the status quo are shown in Table 5. For all tobacco reduction strategies other than taxation alone, costs of the interventions outweighed any associated savings in TB care (Table 6).

**Table 5:** Projected costs and epidemiologic outcomes over 20 years

Outcomes per 1000 persons	Cost *(95% UR*)	TB incidence (*95% UR*)	TB deaths (*95% UR*)	QALYs (*95% UR*)
Status quo	$816,588 *($589,629–$1,144,728)*	18.88 *(14.81–23.92)*	1.83 *(1.12–3.30)*	14,963.00 *(14,935.98–14,966.22)*
Increased taxation	$800,890 *($579,868–$1,120,755)*	18.43 *(14.53–23.30)*	1.76 *(1.11–3.16)*	14,963.66 *(14,937.19–14,966.72)*
Pharmacotherapy and counselling	$955,921 ($726,763–$1,279,070)	18.71 *(14.71–23.68)*	1.80 *(1.12–3.25)*	14,963.25 *(14,936.45–14,966.41)*
Pharmacotherapy, counselling, and mass media	$1,012,812 *($782,975–$1,333,469)*	18.62 *(14.65–23.56)*	1.79 *(1.11–3.22)*	14,963.40 *(14,936.71–14,966.52)*
All [taxation + mass media + pharmacotherapy + counselling]	$981,366 *($764,325–$1,283,894)*	17.72 *(14.06–22.32)*	1.64 (1.03–2.93)	14,964.76 *(14,939.14–14,967.55)*
Costs per person and ICERs—status quo as comparator	Incr. cost vs status quo	Incr. cost per TB case averted	Incr. cost per TB death averted	Incr. cost per QALY gained
Status quo [comparator]	–	–	–	–
Increased taxation	Saves $16 ($8 - $26)	Dominant	Dominant	Dominant
Pharmacotherapy and counselling	$142 *($125–$146)*	$837,464 *($527,624–$1,462,565)*	$4,990,117 *($2,495,571–$8,928,090)*	$542,594 *($259,691–$812,827)*
Pharmacotherapy, counselling, and mass media	$200 *($175–$206)*	$760,387 *($478,141–$1,330,478)*	$4,530,453 *($2,264,728–$8,115,767)*	$492,482 *($235,705–$738,813)*
All [taxation + mass media + pharmacotherapy + counselling]	$181 *($157–$196)*	$142,907 *($78,263–$271,386)*	$852,009 *($398,407–$1,594,941)*	$93,717 *($41,304–$147,677)*
ICERs per person—increased taxation as a comparator	Incr. cost vs Increased taxation	Incr. cost per TB case averted	Incr. cost per TB death averted	Incr. cost per QALY gained
Status quo	$16 *($8–$26)*	Dominated	Dominated	Dominated
Increased taxation [comparator]	–	–	–	–
Pharmacotherapy and counselling	$155 *($139–$164)*	Dominated	Dominated	Dominated
Pharmacotherapy, counselling, and mass media	$212 *($192–$222)*	Dominated	Dominated	Dominated
All [taxation + mass media + pharmacotherapy + counselling]	$180 *($154–$195)*	$256,022 *($151,962–$465,895)*	$1,522,952 *($740,992–$2,789,202)*	$164,369 *($76,252–$252,203)*

*UR* uncertainty range, *ICER* incremental cost-effectiveness ratio, *Incr* incremental

Numbers in parentheses represent 95% UR i.e. uncertainty ranges

**Table 6:** Projected health system and smoking interventions costs over 20 years

Outcomes per 1000 persons	Total cost	TB-related health system cost	Smoking intervention cost
Status quo	$816,588	$816,588	–
Increased taxation	$800,890	$800,890	–
Pharmacotherapy and counselling	$955,921	$810,731	$145,190
Pharmacotherapy, counselling, and mass media	$1,012,812	$807,503	$205,309
All [taxation + mass media + pharmacotherapy + counselling]	$981,366	$776,057	$205,309

We calculated incremental cost-effectiveness ratios (ICERs) comparing the combined strategy with increased taxation alone. The estimated ICERs were (a) $256,022 (95% UR $151,962–$465,895) per TB case averted, (b) $1,522,952 (95% UR $740,992–$2,789,202) per TB death averted, and (c) $164,369 (95% UR $76,252–$252,203) per QALY gained.

### Sensitivity analyses

Tornado diagrams (Additional file 1: Figure S4-S8) show that predicted TB incidence was most affected by the annual probability of reactivation of longstanding LTBI, while TB-related health system costs were primarily influenced by the cost and duration of hospitalization.

As shown in Table 7, increases in the proportion of smokers making quit attempts with pharmacotherapy had some impact on TB incidence and TB-related mortality, but a limited effect on quality-adjusted survival. On the other hand, extending the duration of effect of tobacco reduction interventions had little impact, since most of the TB-related benefit accrued early in the simulation. Indeed, offering pharmacotherapy and counselling annually for the full 20 years was associated with substantial cost increases, but little improvement in TB-related morbidity and mortality (Table 7). Sensitivity analysis of enhanced smoking cessation, either via repeated offers of pharmacotherapy and counselling or by greater uptake of these services, produced considerable reduction in smoking prevalence by the end of the simulation (see Additional file 1: Table S3) with limited changes in TB incidence.

**Table 7:** Sensitivity analysis—projected costs and outcomes per 1000 population over 20 years

Strategy	Cost	TB incidence	TB deaths	QALYs accrued
Pharmacotherapy interventions’ effectiveness lasts 5 years				
Status quo—no new intervention	$816,588	18.88	1.83	14,963.00
Increased taxation	$800,890	18.43	1.76	14,963.66
Pharmacotherapy and counselling	$953,746	18.65	1.79	14,963.33
Pharmacotherapy, counselling, and mass media campaign	$1,009,480	18.52	1.77	14,963.52
Combination of all strategies	$977,469	17.61	1.62	14,964.90
Taxation (one time increase), pharmacotherapy, and mass media continued for 20 years				
Status quo—no new intervention	$816,588	18.88	1.83	14,963.00
Increased Taxation	$800,890	18.43	1.76	14,963.66
Pharmacotherapy and counselling	$2,732,610	18.48	1.77	14,963.48
Pharmacotherapy, counselling, and mass media campaign	$3,444,542	18.27	1.73	14,963.73
Combination of all strategies	$3,047,175	17.36	1.58	14,965.13
Scenario where 50% of smokers attempt quitting and use pharmacotherapy services				
Status quo—no new intervention	$816,588	18.88	1.83	14,963.00
Increased taxation	$800,890	18.43	1.76	14,963.66
Pharmacotherapy and counselling	$1,293,929	17.83	1.66	14,964.42
Pharmacotherapy, counselling, and mass media campaign	$1,288,847	17.63	1.62	14,964.74
Combination of all strategies	$1,226,645	15.87	1.33	14,967.53
Scenario where 75% of smokers attempt quitting and use pharmacotherapy services				
Status quo—no new intervention	$816,588	18.88	1.83	14,963.00
Increased taxation	$800,890	18.43	1.76	14,963.66
Pharmacotherapy and counselling	$1,521,195	16.97	1.52	14,965.59
Pharmacotherapy, counselling, and mass media campaign	$1,512,916	16.68	1.47	14,966.07
Combination of all strategies	$1,433,675	14.46	1.09	14,969.78

Further sensitivity analysis results are presented in Additional file 1.

## Discussion

Our analysis identified limited improvements in tuberculosis-related outcomes with enhanced tobacco reduction, despite the high prevalence of smoking, and well-documented links between tobacco use and TB. These findings reflect the modest effectiveness of existing tobacco reduction approaches, with health gains that become evident only in the longer term. Long-term quit rates remain suboptimal, and more effective smoking prevention and cessation alternatives are clearly needed. In sensitivity analysis, we projected further improvement in TB morbidity with increased uptake of pharmacotherapy for smoking cessation. Hence, more substantial reductions in tobacco use will likely bring both health and economic benefits with respect to tuberculosis in Inuit communities.

Of the strategies we evaluated, taxation involves no direct costs to the health system, so any resulting health gains also save money for the health care system. If increased taxation is indeed as effective in Northern Canada as elsewhere in reducing tobacco consumption, the cost-effectiveness of additional strategies (e.g., the addition of pharmacotherapy and counseling) is likely to be limited with respect to TB-related health outcomes. Taxation has been shown to be effective in reducing smoking prevalence and smoking initiation in younger community members (15–24 years) who may be the most sensitive to tobacco price increases. Smoking prevalence is exceptionally high in this group among Inuit, and likewise, the TB epidemic has consistently involved young adults, making them a key group [14]. This is highly relevant to Inuit communities, where the median age is as low as 21 [22], and where 46% of those who smoke started by age 14 [8, 48]. More generally, the strategies we considered have proven effective in Indigenous settings in Australia, the USA, and Canada [28, 49–52] and are among the measures recommended by the WHO Framework Convention on Tobacco Control [25, 26].

While some authors have projected improvements in tuberculosis morbidity related to reduced tobacco use [5], there are few cost-effectiveness analyses addressing the impact of specific tobacco reduction strategies on TB prevention. Kowada and colleagues evaluated the potential cost-effectiveness of pharmacotherapy in conjunction with screening for latent infection, among TB contacts who smoke [53] and concluded that smoking cessation interventions are likely cost-effective in the management of TB contacts in low-incidence countries.

Our study has several limitations. Most importantly, we did not consider health benefits, QALYs, and cost savings of reduced tobacco use beyond the TB context, which are of course well documented in other clinical settings [54–57]. In fact, integration of other benefits would further strengthen the argument for investment in smoking cessation and prevention. Though this analysis found modest improvements in TB morbidity, we estimated a 10% absolute reduction in smoking prevalence with the combined strategy. Of note, there is also evidence that smoking cessation interventions improve quit rates and quality of life among persons with TB [58–62]. On the other hand, there are lags between smoking cessation and many associated health benefits (e.g., reductions in lung cancer and coronary artery disease risks). We did not model such lags with respect to TB-associated risks, as at present there are no firm data in this regard. The systematic review and meta-analysis by Lin and colleagues identified some studies which indeed suggested persistent elevation of TB risks in former smokers [6]. This would further limit short-term improvements in TB-related health outcomes related to reduced tobacco use. The same may be true for concomitant alcohol use, which was a potential unadjusted confounder in some studies included in that meta-analysis.

While we had high-quality local data for TB-related health care costs, some cost estimates for tobacco reduction strategies had to be obtained elsewhere, as there were no such data from the Canadian Arctic. It is also possible that price elasticity for tobacco products may differ in the Arctic setting, meaning that tax increases may have greater or lesser effects than observed elsewhere. However, our base case estimate for price elasticity was obtained from a comprehensive review [63],

It is essential that any strategy to reduce tobacco use be concordant with the community’s experience, culture, and values. Because of limited information, many model inputs unfortunately did not reflect Inuit-specific data or experience, though scenarios were vetted by community partners. If tobacco and TB reduction efforts are to be successful, they must reflect community-based programs to address upstream determinants, informed by members’ experience and values [64]. Such programs may provide effective adjuncts or alternatives to TB-specific technical interventions, since they focus on prevention rather than enhanced diagnostic capacity. The relative isolation of many Northern communities also highlights the potential role of non-technical approaches to TB prevention. However, policymakers must also recognize that the benefits of reduced tobacco use for TB prevention will likely become apparent only very gradually.

## Conclusions

Smoking prevention and cessation strategies are projected to have a modest impact on TB-related morbidity in Inuit communities in the short to medium term, but some may be cost saving. However, even small reductions in tobacco use may strengthen TB prevention efforts and provide gains in other health outcomes. Although we focused on a very specific Northern setting, our methods and findings are relevant to other areas where both tobacco use and TB are highly prevalent [65]. Other risk factors such as alcohol use, nutrition and food security, and housing conditions could also be considered [66–68].

## Additional file

Additional file 1: Supplemental Methods and Results. (DOCX 849 kb)

## Abbreviations

CDC: Centers for Disease Control and Prevention
CTADS: Canadian Tobacco, Alcohol and Drugs Survey
CXR: Chest X-ray
ICER: Incremental cost-effectiveness ratio
LTBI: Latent TB infection
NRT: Nicotine replacement therapy
PSA: Probabilistic sensitivity analysis
QALY: Quality-adjusted life year
TB: Tuberculosis
TST Test: Tuberculin skin test
UR: Uncertainty range
WHO: World Health Organization
