# Supplementary material for: The potential impact and cost-effectiveness of tobacco reduction strategies for tuberculosis prevention in Canadian Inuit communities
Source: BMC Med. 2019 Feb 4;17:26. doi: 10.1186/s12916-019-1261-5 (PMC6360759; doi:10.1186/s12916-019-1261-5)
Supplement: Supplementary file 1 — Supplemental Methods and Results. (DOCX 849 kb) [file 12916_2019_1261_MOESM1_ESM.docx]

**Additional file 1: The potential impact of tobacco reduction strategies on tuberculosis in Canadian Inuit communities**

[Methods 2](#_Toc522113744)

[Dynamic model 2](#_Toc522113745)

[Overview 2](#_Toc522113746)

[Model structure 2](#_Toc522113747)

[SLIR model states 3](#_Toc522113748)

[Model calibration and validation 6](#_Toc522113749)

[Summary 6](#_Toc522113750)

[Phase I: From seeding to equilibrium 7](#_Toc522113751)

[Seeding (Initial conditions) 7](#_Toc522113752)

[Equilibrium (Pre-1948 model) 7](#_Toc522113753)

[Phase II: 1948-1697 8](#_Toc522113754)

[Phase III: Introduction of diagnosis and treatment of LTBI & active TB (1967-2000) 8](#_Toc522113755)

[Phase IV: End of dynamic modeling – rising TB incidence (2000-2017) 8](#_Toc522113756)

[Cost effectiveness analysis (using Decision Analysis) 11](#_Toc522113757)

[Overview 11](#_Toc522113758)

[Model Structure 12](#_Toc522113759)

[Data inputs 12](#_Toc522113760)

[TB Pathogenetic and Epidemiologic parameters: 12](#_Toc522113761)

[TB Program parameters - Standard of Care for management of TB in Northern Inuit Communities 13](#_Toc522113762)

[Tobacco reduction Strategies: 13](#_Toc522113763)

[Results 18](#_Toc522113764)

[SLIR model equations 24](#_Toc522113765)

[Non-Smoker Stratum 24](#_Toc522113766)

[TB Health States: 24](#_Toc522113767)

[Difference Equations: 25](#_Toc522113768)

[Initial Conditions: 25](#_Toc522113769)

[Smoker Stratum 26](#_Toc522113770)

[TB Health States: 26](#_Toc522113771)

[Difference Equations: 26](#_Toc522113772)

[Initial Conditions: 27](#_Toc522113773)

[Parameter Values 27](#_Toc522113774)

[Pathogenetic Parameters (Non-Smokers): 27](#_Toc522113775)

[Pathogenetic Parameters (Smokers): 28](#_Toc522113776)

[Other Parameters (Common to Non-Smokers & Smokers): 28](#_Toc522113777)

[References 29](#_Toc522113778)

# Methods

## Dynamic model

### Overview

We modeled the tuberculosis (TB) epidemic with a susceptible-latent-infectious-recovered (SLIR) TB transmission model to predict key measures of the TB epidemic in the Inuit community such as annual risk of TB infection (ARI), incidence of active TB, and TB-related mortality. The model output was then used to provide starting distributions for TB-related health states, for a cost-effectiveness analysis of the impact of reducing tobacco use on TB related outcomes using decision analysis modeling.

The major events and time covered by the dynamic model were: (a) the TB epidemic in Canadian Inuit communities, before the advent of antibiotics; (b) the introduction of certain changes in living conditions as well as antibiotic treatment, and their impact on the epidemic; and (c) a more recent period of TB prevention and care measures. We modelled the historical phases of the epidemic based on published data and calibrated unknown parameters against observed historical data. At the end of the period covered by the dynamic model we extrapolated observed trends in the first decade of the 21^st^ century, a period that includes several TB outbreaks in Inuit communities.

### Model structure

A SLIR transmission model structure was used, with difference equations representing the transition of community members from one TB related health state to another, while accounting for smoking behavior. The model included a stratum for smokers (where risks of acquiring TB infection and of progression from latent to active TB were increased) and a stratum for non-smokers, with possible transition between the two strata, as well as transitions between the various TB-related health states. Transition between TB states and smoking strata occurred at rates obtained from the literature (see figures 1 and 2) and compartments were populated using the difference equations listed at the end of this appendix.

#### SLIR model states

The model had eight TB state compartments grouped in two strata (smokers and non-smokers) (figure 2). (1) A ***susceptible*** state with individuals who were never infected who could become newly infected at an annually risk of infection that was higher in the smoking stratum that in the non-smoking stratum.

Latent infection included (2) ***fast progressing latent TB infection (fast LTBI)*** where only newly infected individuals could enter this compartment. From this compartment individuals could be re-infected or progress toward (3) ***longstanding LTBI (termed slow LTBI in the model).*** Since individuals could potentially have long standing LTBI without reactivating to active TB, we considered that some such individuals could experience (4) ***LTBI re-infection***, reactivate to the active TB (infectious) compartment or return to the long standing LTBI compartment. For reinfected persons, their rate of reactivation toward infectious disease was lowered due to immunity conferred by previous latent infection.

(5) The ***Infectious state*** (active TB) was populated by transitions from the three latent infection compartments listed above. Infected individuals could (6) ***recover following treatment*** or (7) ***recover spontaneously without treatment***. Individuals who had recovered from active TB could also return to the infectious state via relapse. Recovered individuals could also be reinfected.

The cohort size was assumed to be stable throughout the dynamic simulation; to achieve this, the combined death rate was equal to the birth rate (i.e. number of individuals entering the model).

**Table S1: SLIR model parameters**

| Parameter | Parameter description | Parameter value* | | |
| --- | --- | --- | --- | --- |
|  |  | **1948** | **2000** | **2017** |
| ARI | annual risk of infection; calculated using b | 25 | 0.49 | 0.95 |
| p | rate of primary progression from LTBI to active TB | 0.055^†^ | 0.037 | 0.045 |
| v | annual reactivation rate from long standing LTBI to active TB | 0.001^‡^ | 0.0005 | 0.0005 |
| t | transition rate from latent fast to latent slow | 0.3^‡^ | 0.3 | 0.3 |
| z | death rate of untreated TB | 0.2^‡^ | 0.2 | 0.2 |
| d | natural cure rate | 0.25^‡^ | 0.25 | 0.25 |
| tx_rate | active TB treatment rate | 1^‡^ | 1 | 1 |
| d_rate | background mortality rate | 0.023^‡^ | 0.016 | 0.019 |
| prot_ltbi | sensitivity and efficacy of LTBI treatment | 0.4833^‡^ | 0.4833 | 0.4833 |
| im | immunity conferred by primary infection | 0.55^†^ | 0.55 | 0.55 |
| cd | diagnosis rate for active TB | 0^‡^ | 0.9 | 0.9 |
| cd_latent | diagnosis rate for latent disease | 0^‡^ | 0.03 | 0.03 |
| txs | probability of active treatment completion and success | 0.97^‡^ | 0.97 | 0.97 |
| zd | death rate under active TB treatment | 0.042^‡^ | 0.042 | 0.042 |
| b | beta, contact rate | 5^†^ | 1.84 | 3.63 |
| v_SponRec | reactivation rate after spontaneous recovery | 0.025^‡^ | 0.025 | 0.025 |
| v_ActiveTx | reactivation rate after active treatment | 0.015^‡^ | 0.015 | 0.015 |
| 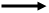 | Model entry and exit flows of individuals. |  |  |  |
| \| 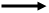 \| \| --- \| | Arrows represent flows between the compartments and correspond to transition between different TB health states according to rates applied to the cohort in respective compartments. |  |  |  |

***** For parameter values that change over time, see SLIR model equations at the end of the appendix for exact trends. All values listed in this table pertain to non-smokers. Values for smokers were obtained by multiplying these values by their respective estimates of smoking effect from literature, as described in main manuscript.
^†^ Calibrated against observed ARI and incidence rate using the dynamic model; see detailed description below.
^‡^ Values based on those from literature; references included at the end of the main text and supplementary text.

## Figure S1: Equilibrium model (pre 1948)

##
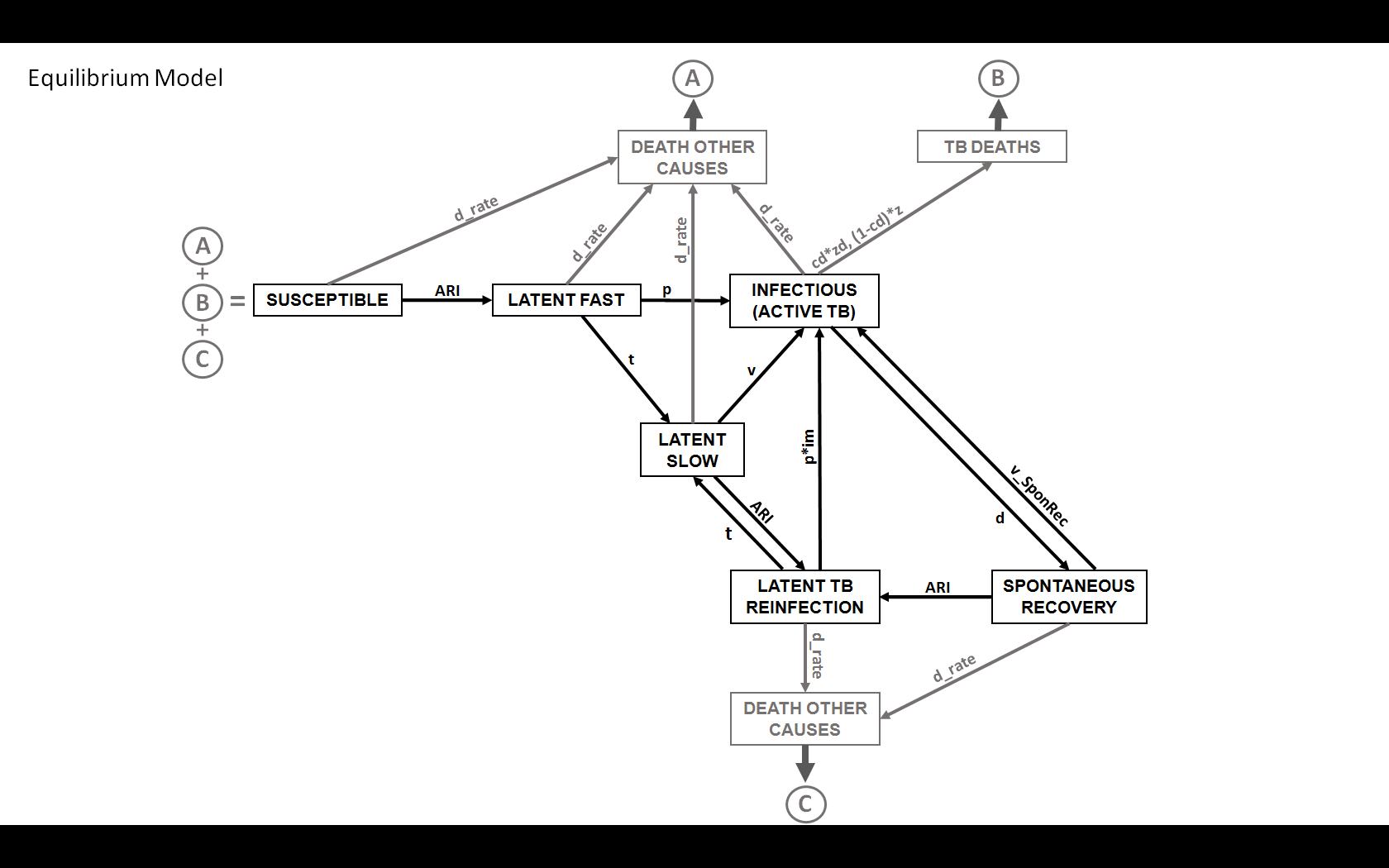


**Figure S2: SLIR model with diagnosis and treatment of LTBI & active TB (post 1948)**


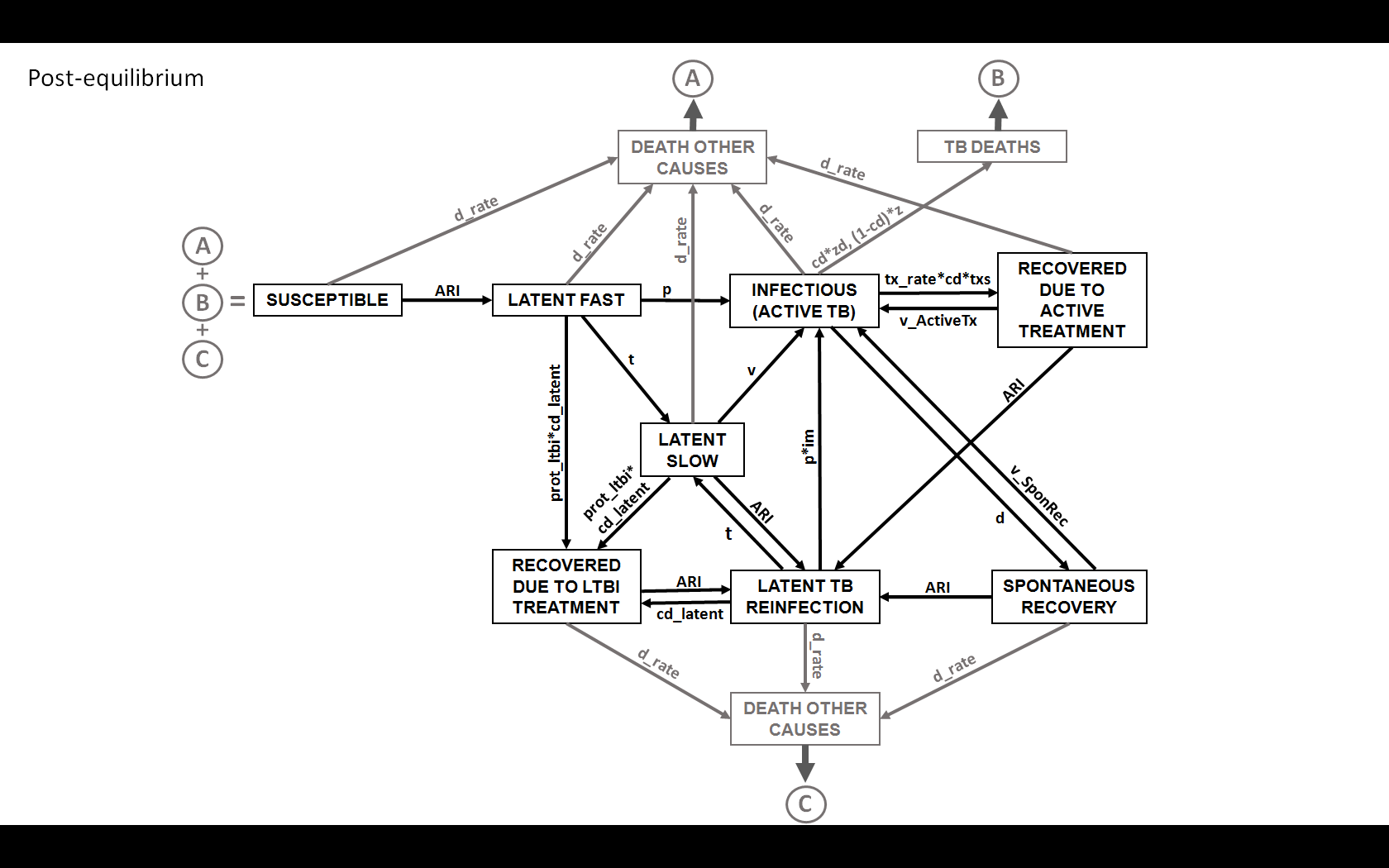


#### Model calibration and validation

##### **Summary**

Four different phases capturing the history of the TB epidemic among Canadian Inuit were simulated.

The first phase involved seeding of the model which corresponds to the introduction of an infectious TB case into a fully susceptible population; followed by simulation over several decades to attain equilibrium where TB incidence and smoking prevalence were stable.^1^ We used observations from this period to estimate the beta parameter (the rate of effective contact between infectious and non-infectious persons per unit of time). We considered that the first phase ended in 1948.

The second phase of the simulation covered the period between the year 1948 and 1967. In this period TB incidence is known to have declined and smoking prevalence was documented to be stable.^1^ Antibiotic use was limited. However, living conditions began to improve and this was captured by reduction in the beta parameter ^2^. In order to calibrate the rate at which the beta declined, we used historical observed TB incidence and ARI data from the Canadian Eastern Arctic ^3, 4^. The first two phases marked a pre-treatment era that ended in 1967.

The third and fourth phases of the dynamic model covered a period of decline in TB incidence and smoking prevalence from 1967 to 2000, and then finally a period of increasing TB incidence from 2000-2017. It is early in the third phase that current, standard TB related interventions (i.e. active case finding, smear and culture-based diagnosis, modern drug treatment and INH treatment of latent infection) were widely adopted. The model was calibrated during this period of declining TB incidence (1967- 2000) using observed incidence and ARI ^4^. We continued the calibration during 2000-2017, a period that included outbreaks of TB in Nunavut and Nunavik ^5, 6^. At the end of the dynamic simulation in 2017, key model outputs such as population distribution in each TB-related health state, and other model parameters such as the annual risk of infection were stratified by smoking status. These values were then used as baseline parameters for the Markov model beginning in 2017.

##### **Phase I: From seeding to equilibrium**

###### Seeding (Initial conditions)

An infectious individual was introduced into a population of fully susceptible individuals. The cohort was spread across the two strata with a smoking prevalence of 90%.^7^

###### Equilibrium (Pre-1948 model)

During this period, we simulated the natural history of TB morbidity and mortality in the cohort until it reached equilibrium. Diagnosis and treatment of LTBI and active TB were non-existent. Indeed, the aim was to simulate the natural history of TB before any intervention in order to assess key parameters of the epidemic unaltered by public health measures (see Figure S1 above for a simplified schematic). We calibrated three parameters against observed annual risk of infection (ARI) and TB incidence; (1) contact rate (**b**), (2) progression rate (**p**), and (3) immunity conferred by previous infection (**im**). These parameter values and the ensuing cohort distribution across the various model states were used as baseline in a post-1948 SLIR model.

##### **Phase II: 1948-1967**

This was a period of documented decline in the contact rate due to changes in living conditions.^3^ This led to decreases in TB incidence which were not due to TB program improvement as no new interventions were implemented. Indeed, during this period, some Inuit communities suffered forced resettlement. The beta (contact rate) and progression rate declined accordingly and were calibrated using published TB incidence in Inuit communities.^3, 4^

##### **Phase III: Introduction of diagnosis and treatment of LTBI & active TB (1967-2000)**

Diagnosis and treatment of LTBI and active TB were widely introduced in 1967. To capture this change of TB management in our cohort, some structural changes were integrated into the model; treatment related compartments were added and corresponding transition rates were introduced, with the assumption that diagnosis and treatment rates increased in linear fashion to reach a plateau.

The Inuit-specific annual rate of smoking cessation was estimated from observed decreases in smoking prevalence.^7-13^ ^14-16^ As smoking prevalence decreased slowly but consistently, we assumed that adult smokers could transition to non-smoking states, but not the reverse.

We considered a continuous decline of the beta parameter from 1948-1988 to capture improved standards of living. Starting in 1988, however, beta reached a plateau, thereby symbolizing the end of living condition improvements. In terms of the Inuit population timeline in Canada, this corresponds to the period when life expectancy stabilised after a steady increase.^17^ Consequently, from this point until the year 2000, further decrease in the ARI was considered to be solely due to the decrease in active TB cases from treatment and smoking cessation. Then, starting in 2000, beta began to increase; details are in the following section.

##### **Phase IV: Rising TB incidence (2000-2017)**

In more recent years, the rate of decline in smoking prevalence has slowed, and the incidence of TB has increased.^1,5,6^ To capture this trend, fewer individuals transitioned from smokers to non-smokers, and some model parameters were re-calibrated – namely, the beta and progression parameters. Both beta and the progression rate were set to increase after 2000, generating a rise in both ARI and TB incidence in order to match the observed increase in TB incidence. An increase in beta alone was insufficient to reproduce the observed increase in TB incidence.

**Calibration Details:**

The model was calibrated against two observed outcomes: ARI and TB incidence rate (see Supplemental Table S4 for observed raw data). During Phase I, the three parameters that were calibrated were the beta, the progression parameter and immunity parameter. To calibrate, first observed values for both incidence rate and ARI were linearly approximated by a trend line, and values between observed data points were interpolated. Data sets were then imported into Berkeley Madonna (Version 8.3.23.0, University of California, 2015). The program’s “Curve Fit” calibration tool was used to fit model parameters, based on a Root Mean Squared (RMS) deviation method. In order to refine estimates, the program’s “slider” tool was used to further adjust parameters so that the modelled ARI and incidence rate could be brought closer to their observed values, while making sure that parameters stayed within reasonable bounds, which were informed by those specified in the literature. This procedure was performed for both the equilibrium model (pre-1948) and the post-equilibrium model (1948-67). Below is more detail on the three parameters that were calibrated.

*(1) Contact Rate (b):* The beta parameter began at a value of 5, and declined until a pivotal time point in the year 1988 (t = 40 in the model). In the late 1980s, life expectancies in the North began to plateau before experiencing a slight decline in recent years.^17^ Accordingly, the model was calibrated so that beta also plateaued at this time at a value of 1.84, and began increasing in the year 2000 (t = 52 in the model). This is another pivotal time point where observed ARI and incidence rates began increasing in the North. In both cases where the parameter’s value changed over time, linear or exponential trends were used, according to the best fit against observed ARI and incidence rates. The trend is described by the following equation:

$$b=if t<40 then 5e^{-0.025t} else if 40\leq t<52 then 1.84 else 1.84e^{0.04\left( t-52 \right)}$$

## *(2) Progression Rate (p)* Similarly, this parameter was calibrated so that modelled incidence rate and ARI followed trends in their observed values. It began at a value of 0.055 (for non-smokers) and declined linearly until a pivotal time point in the year 2000 when the observed incidence rate began to increase. Consequently, in the model the progression rate reached a plateau at 0.045 in 2000. The trend is described by the following equation:

$p= if t<52 then 0.055e^{-0.0075t} else 0.0372+0.00045(t-52)$. For those in the smoking stratum, values of this parameter are multiplied by the estimate of effect for smoking on disease progression.

## (3) Immunity (im)

This parameter reflects the immunity rate conferred by primary infection, and was calibrated in the equilibrium model against the observed ARI and incidence rate in 1948. As such, the value obtained was 0.55; unlike the contact and progression rates, this value remained constant throughout the duration of the dynamic model.

**Additional parameters informed by the literature that changed over time:**

In addition to those model parameters that were calibrated as described above, three additional parameters changed over time, in order to be consistent with the literature. More details on how and when these values changed follow below:

*(1) Background Mortality Rate (d_rate_)*
This parameter was based on age-specific life expectancy data in Northern Canada from the Canadian Human Mortality Database, from 1950-2011.^17^ Background mortality was calculated as 1/life expectancy and was used to generate the parabolic approximation seen in the model. It was parabolic because in recent years, life expectancy has shown a slight decline in this region. The trend is described by the following equation: $d_{rate}=0.000005t^{2}-0.0004t+0.023$

*(2) Reactivation (v)*
The reactivation parameter started at 0.001 (for non smokers) and declined linearly until the year 2000 at which point it reached a plateau of 0.0005, reflecting values specified in the literature.^18-20^ The trend is described by the following equation: $if t \leq52 then 0.001-0.0000096t else 0.0005.$ For those in the smoking stratum, values of this parameter are multiplied by the estimate of effect for smoking for reactivation.

*(3) Case detection (cd and cd_latent_)*
A common pivotal time point for case detection of both active and latent TB was the year 1968 –when TB treatment was implemented in the Canadian Arctic, according to Grzybowski.^3^ After this time point, we assumed that case detection increased as reflect in observed ARI and incidence rate. In order to bring predicted data in-line with observed incidence rate and ARI trends, we assumed that case detection for active TB reached 90% in 1978 and remained constant from that year onwards. Similarly, the annual probability of detection of latent infection reached 3% in 1993, and remained constant form that year onwards, concordant with known low rates of LTBI screening outside the contact investigation setting. The trends are described by the following equations:

$$cd_{latent}=if t<20 then 0 else if 20\leq t<45 then 0.0012\left( t-20 \right) else 0.03$$

$$cd= if t<20 then 0 else if 20\leq t<30 then 0.08\left( t-20 \right)+0.1 else 0.9$$

## Cost effectiveness analysis (using Decision Analysis)

### Overview

With decision analysis, we simulated current tobacco control measures using outputs from the dynamic model estimates for 2017; we compared current practice to other potential tobacco control interventions moving forward. Key outputs from the dynamic model included baseline population distribution in the different TB-related health states, annual risk of infection, progression and immunity rates. Other parameters came from the published literature, using Inuit specific data wherever possible.

### Model Structure

Health states in the Markov decision analysis model paralleled the SLIR model compartments including: Susceptible, Latent TB Infection, Infectious (Active) TB, and Recovered states (Figure 2), all stratified by smoking status. Two health states related to mortality were also included: death due to TB, and death from other causes. The Markov cycle length was one year. In addition to transitions between the TB-related health and treatment states, the model included an annual probability of smoking cessation. Outcomes assessed included TB incidence, TB related mortality, quality adjusted life years (QALYs) and costs from the health care system perspective.

### Data inputs

#### TB Pathogenetic and Epidemiologic parameters:

As described above, several pathogenetic and demographic inputs were derived from the dynamic epidemiologic model. These included the initial distribution of the population across the various health states (by both TB and smoking status), the initial annual risk of infection, and the probability of progression to active TB during the first 2 years after newly acquired infection (4.5% annually for 2 years for non smokers). Other key input values were obtained from the literature. For example, the probability of reactivation to active TB beyond 2 years after initial infection was 0.1% per year for non smokers which declined in the dynamic model until it reached 0.05% per year ^18-20^ , The value of 0.05% was then used throughout the decision analysis model. For untreated active TB, the probabilities of spontaneous recovery and death were 25% ^1^ and 19% ^21,22^ respectively. Because of the relatively high annual risk of infection, we included a possibility of re-infection after LTBI or active TB treatment. However, previous infection confers partial protection against progression to active TB in case of repeat infection.^23,24^

#### TB program parameters - Standard of care for management of Active TB and LTBI in Northern Inuit Communities

Current guidelines in Nunavut recommend using a rapid automated PCR test (Xpert®MTB/RIF) for the diagnosis of active TB ^25^ and TB treatment should occur in an outpatient setting, with occasional transfers (4%) south to Ottawa for patients with complications and/or complex medical situations. In our models, we assumed that 90% of persons with active TB were diagnosed and treated, with 100% treatment completion.^26^ For latent TB infection (LTBI), we assumed that 25% of persons infected would be diagnosed and of those diagnosed, 47% started on treatment for LTBI.^27^ For those who start treatment, the probability of LTBI treatment completion was 70.8% based on Nunavut program data.^27^

#### Tobacco reduction strategies:

*Pharmacotherapy key parameters* - The proportions of smokers who made quit attempts and used pharmacotherapy were derived from the Canadian Tobacco Use Monitoring Survey (CTUMS) and the Canadian Tobacco, Alcohol and Drugs Survey (CTADS).^28^ 14.1% of smokers aged ≥18 years classified as current or former smokers made a quit attempt and used a prescription medication such as Bupropion or Varenicline, supplemented by nicotine patch or gum.^29^ An additional 5.7% of current smokers made a quit attempt following the mass media intervention, since media campaigns focused on smoking cessation were expected to increase quit attempts by 40%.^34^

a) A systematic review by Stead et al. showed that individuals receiving pharmacotherapy coupled with counselling were 87% more likely to quit commercial tobacco smoking than smokers without a smoking cessation intervention [risk ratio (RR): 1.60, CI: 1.53-1.68, p<.001 for pharmacotherapy only plus an additional effect of counselling/behaviour support as an adjunct to pharmacotherapy for smoking cessation RR: 1.17, CI: 1.11-1.24, p<.001 for counselling].^30-32^ Hence, the annual reduction in net smoking prevalence for this intervention was estimated to be 1.5% [i.e. 0.8% *(1.60*1.17)], where 0.8% is the background rate of smoking cessation. The effect of this once off intervention was estimated to last three years.^33, 34^

b) A systematic review by Marley et al. suggested that individuals receiving pharmacotherapy plus counselling in communities receiving mass media interventions were twice as likely to quit commercial tobacco smoking than those who received usual care [RR: 2.36, CI: 1.01-5.5, p=0.047].^34^ The annual reduction in smoking prevalence for this once off intervention was estimated to be 1.9% [i.e. 0.8% *(2.36)], where 0.8% is the background rate of smoking cessation. The effect of this once off intervention was estimated to last three years.^33, 34^

*Key taxation parameters* - A meta-analysis of 86 articles examined the price elasticity of demand for tobacco products and estimated a mean price elasticity of -0.48 [i.e. 10% increase in price is associated with a 4.8% decrease in consumption].^36^ A SimSmoke simulation notes that 25% price increase leads to 7% reduction in smoking prevalence within three years and increases over time to 14%.^34, 37^ The effect of tax increases on smoking prevalence was further illustrated by a reduction in tobacco purchases for two years following the 2012 tax increases in Nunavut. This was cited by the Nunavut department of finance as the rationale for an additional tobacco tax increase in 2017.^38^

*Combined strategy key assumptions* – The annual reduction in net smoking prevalence for all strategies combined was obtained from a multiplicative effect of [annual reduction in smoking prevalence due to taxation] and [RR for smoking cessation of pharmacotherapy plus mass media].

**Table S2. Sensitivity analysis - Distributions of input parameters**

| Description | Distribution | Point estimate* | Range |
| --- | --- | --- | --- |
| Annual risk of TB infection among non-smokers | Beta | 0.006 | [0.005 – 0.008] |
| Annual risk of TB infection among smokers | Beta | 0.01 | [0.009 – 0.015] |
| Immunity from previous disease | Beta | 0.55 | [0.4 - 0.7] |
| Probability of an individual evaluated for TB in a remote community being sent to Iqaluit for further TB related work-up* | Beta | 0.04 | [0.04 - 0.05] |
| Probability of coming back for TST reading* | Beta | 0.86 | [0.8 - 0.9] |
| Probability of completing active TB treatment* | Beta | 0.95 | [0.94 - 0.96] |
| Probability of completing latent TB treatment * | Beta | 0.71 | [0.531 - 0.885] |
| Probability of diagnosis of TB disease among individual with active disease* | Beta | 0.90 | [0.8 - 1] |
| Probability of having adverse event from latent TB treatment* | Beta | 0.003 | [0.001 - 0.005] |
| Probability of having adverse event from TB treatment* | Beta | 0.05 | [0.01 - 0.1] |
| Probability of living in a remote community without a hospital* | Beta | 0.21 | [0.16 - 0.26] |
| Probability of LTBI diagnosis* | Beta | 0.25 | [0.2 - 0.9] |
| Probability of producing suitable sputum samples given CXR abnormality* | Beta | 0.82 | [0.747 - 0.896] |
| Probability of progression of recently acquired LTBI to TB disease for non-smoker* | Beta | 0.045/year x 2 years | [0.034 -0.056] |
| Probability of reactivation of old-standing LTBI to TB disease for non-smoker* | Beta | 0.0005/yr | [0.0004 – 0.0007] |
| Probability of cure among those who complete treatment for active TB* | Beta | 0.95 | [0.9 - 1] |
| Probability of cure following complete treatment for latent TB* | Beta | 0.90 | [0.875 - 0.925] |
| Probability of spontaneous recovery from active TB* | Beta | 0.22 | [0.2 - 0.3] |
| Probability of TB-related death during TB treatment* | Beta | 0.02 | [0.012 - 0.032] |
| Cost of 7 outpatient clinic visits during isoniazid treatment † | Triangular | $609.23 | [$456.92 - $761.54] |
| Cost for transfer of critically ill patients to the south | Triangular | $1501.62 | [$1127.26 - $1880.51] |
| Cost of a chest X-ray * | Triangular | $70.87 | [$53.15 - $88.59] |
| Cost of a day of hospitalisation | Triangular | $2588.72 | [$1944.06 - $3241.15] |
| Cost of a spontaneous sputum production | Triangular | $3.67 | [$2.76 - $4.59] |
| Cost of an analysis of a positive sputum | Triangular | $84.17 | [$62.51 - $106.27] |
| cost of analysis of a negative sputum | Triangular | $30.57 | [$22.92 - $38.2] |
| Cost of drug for DOT | Triangular | $640.84 | [$480.63 - $801.05] |
| cost of follow up following treatment Active treatment initiation | Triangular | $147.55 | [$115.47 - $192.46] |
| Cost of follow-up clinic visit after tuberculin test † | Triangular | $59.31 | [$44.48 - $74.14] |
| Cost of induced sputum production † | Triangular | $100.89 | [$75.67 - $126.11] |
| Cost of tuberculin skin test † | Triangular | $18.98 | [$14.24 - $23.73] |
| Cost of return flight to Nunavut after treatment in Ottawa † | Triangular | $10427.45 | [$7820.59 - $13034.31] |
| Hourly wage for a doctor only for non-remote community † | Triangular | $69.31 | [$51.98 - $86.64] |
| Cost of initial TB assessment in a remote community : Nurse fee | Triangular | $27.13 | [$18.81 - $31.36] |
| Cost of Isoniazid, 9 months supply † | Triangular | $182.01 | [$136.51 - $227.51] |
| Cost of major adverse reaction to Isoniazid † | Triangular | $15561.57 | [$11671.18 - $19451.96] |
| Nurse assessment wage for 20-min visit | Triangular | $27.13 | [$18.81 - $31.36] |
| Relative risk of progression to active TB due to smoking | Triangular | 1.50 | [1.26 - 1.74] |
| Relative risk of death among smokers compare to no smokers | Triangular | 2.60 | [1.8 - 3.6] |
| Relative risk of LTBI infection due to smoking | Triangular | 1.90 | [1.6 - 2.3] |
| Utility score for individual with active disease treated | Triangular | 0.85 | [0.8 - 0.9] |
| Utility score for individual with active disease untreated | Triangular | 0.68 | [0.65 - 0.7] |
| Utility score for individual with latent disease treated | Triangular | 0.97 | [0.95 - 1] |
| Utility score for individual with latent disease untreated | Triangular | 1.00 | [0.99 - 1] |
| hospitalisation days at initiation | Uniform | 14.00 | [10 - 18] |
| Number of days of hospitalisation in case of suspicion of active disease before the diagnosis results | Uniform | 3.00 | [2 - 4] |

* All probabilities are one-time, unless otherwise specified

† Range: ±25% from the point estimate

**Table S4. Observed TB incidence rate and ARI used for model calibration (raw data)**

| Time Point in the Model | Corresponding Year | Observed Annual Risk of Infection (ARI) | Observed Incidence Rate (per 100,000 person-years) |
| --- | --- | --- | --- |
| 0 | 1948 | 25 | 2900 |
| 17 | 1965 |  | 1490 |
| 23 | 1971 | 3.5 |  |
| 26 | 1974 | 1.75 |  |
| 52 | 2000 |  | 172.9 |
| 53 | 2001 |  | 139.5 |
| 54 | 2002 |  | 93.7 |
| 55 | 2003 |  | 23.9 |
| 56 | 2004 | 0.33 | 107.2 |
| 57 | 2005 |  | 148.4 |
| 58 | 2006 |  | 155.8 |
| 59 | 2007 |  | 98.7 |
| 60 | 2008 |  | 185 |
| 61 | 2009 |  | 168.7 |
| 62 | 2010 |  | 299.8 |
| 63 | 2011 |  | 216.4 |
| 64 | 2012 |  | 230.5 |
| 65 | 2013 |  | 143.3 |
| 66 | 2014 |  | 229.6 |
| 67 | 2015 |  | 119.2 |

**Figure S3: CEA model schema**


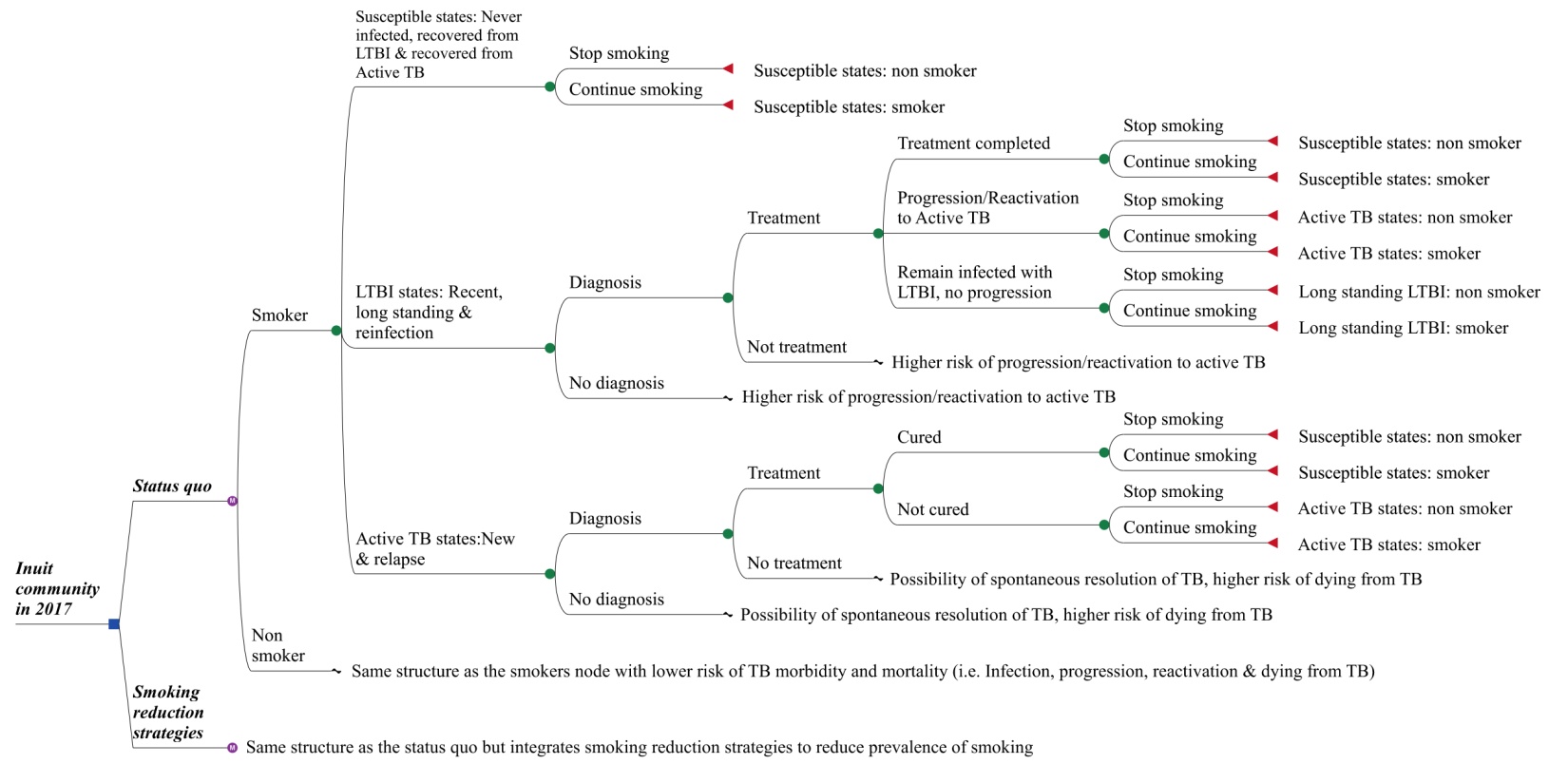


# Results

**Figure S4:** **Univariate sensitivity analysis (cost per person in multifaceted strategy)**


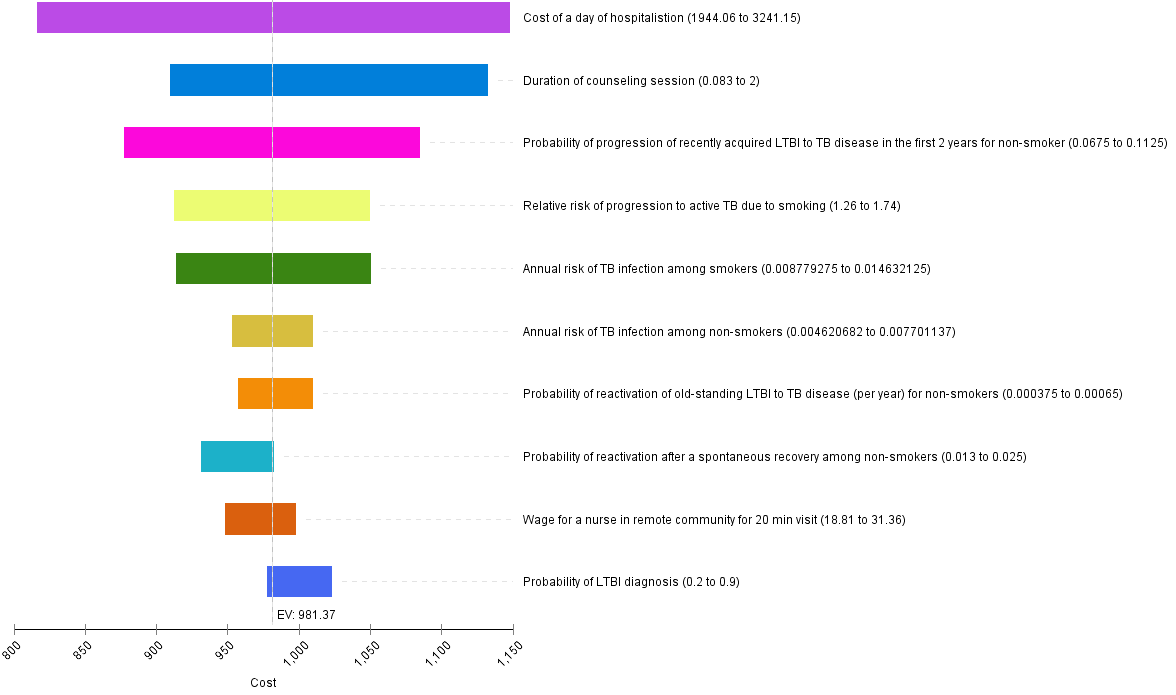


## Figure S5: Univariate sensitivity analysis (TB incidence per person in multifaceted strategy)


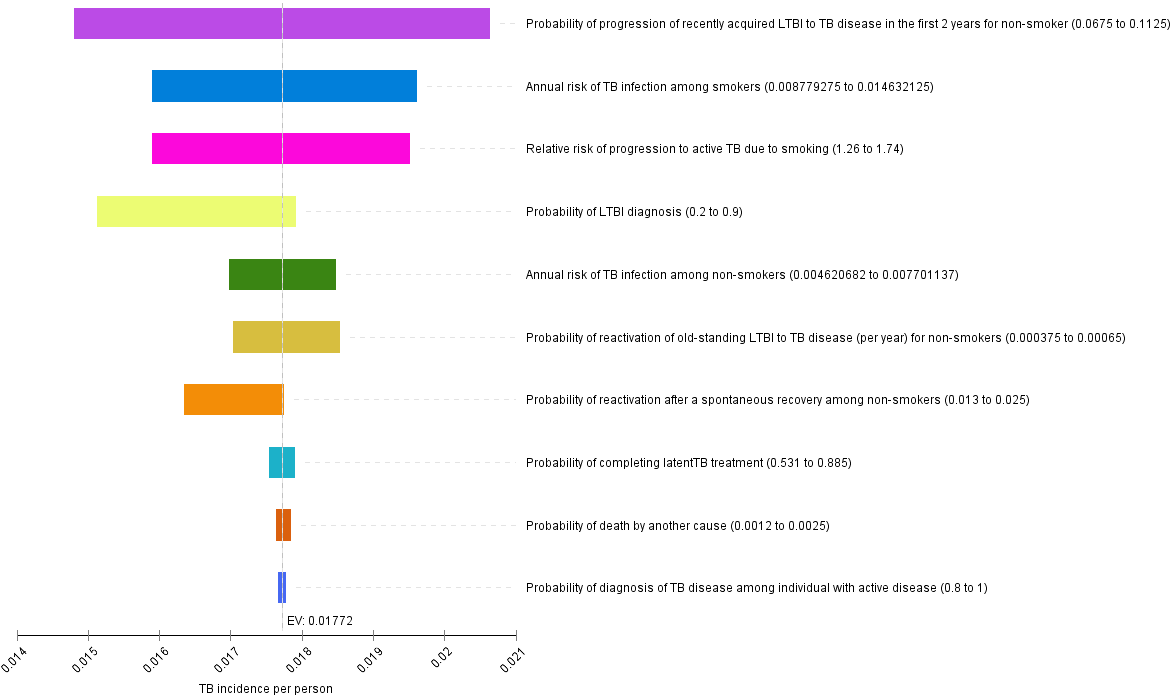


**Figure S6: Univariate sensitivity analysis (TB death per person in multifaceted strategy)**


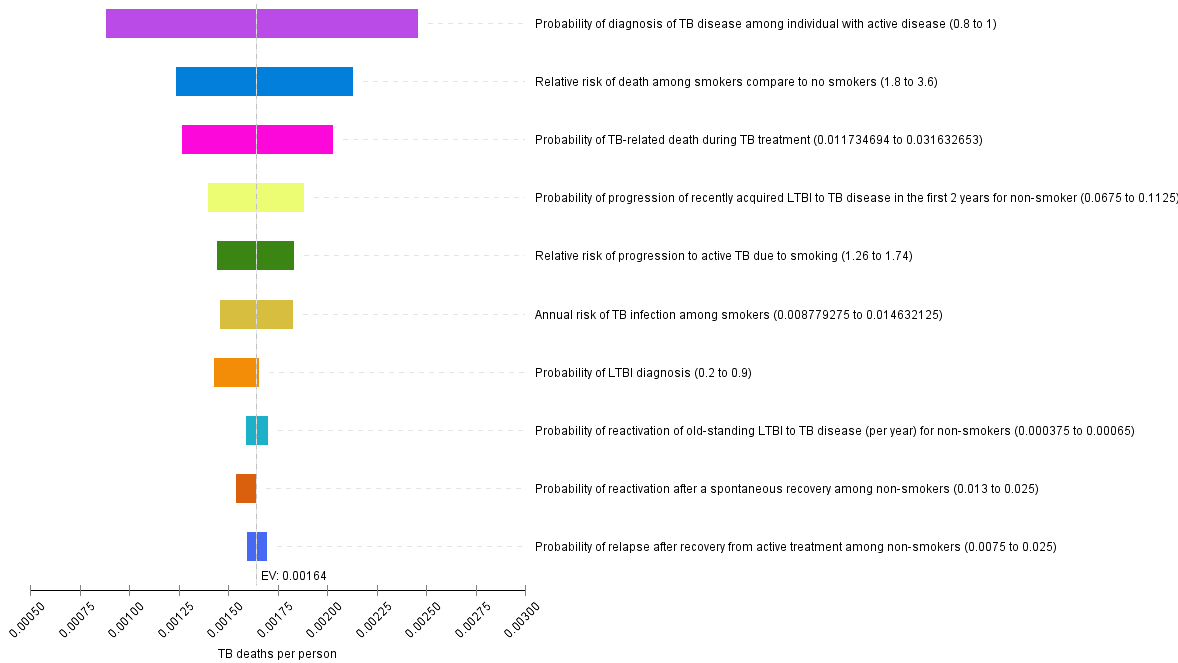


**Figure S7:** **Sensitivity analysis (Incremental cost per TB cases averted: multifaceted strategy vs. status quo)**


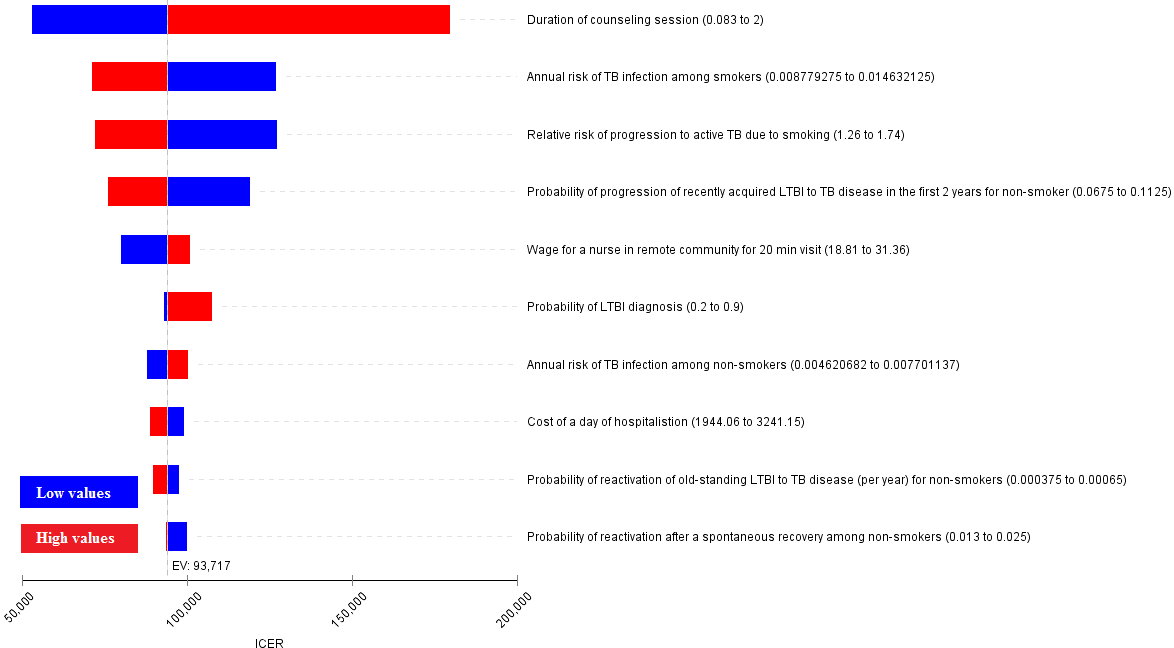


**Figure S8: Sensitivity analysis (Incremental cost per TB cases averted: multifaceted strategy vs. increased tation)**


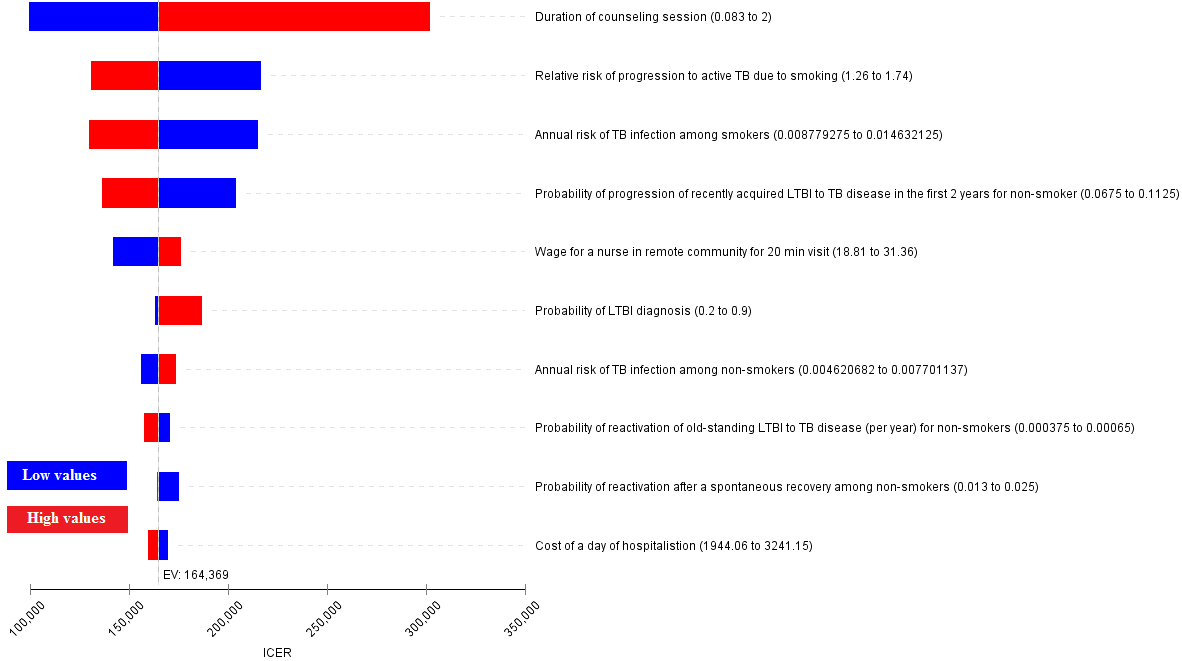


**Figure S9: Probabilistic sensitivity analysis**


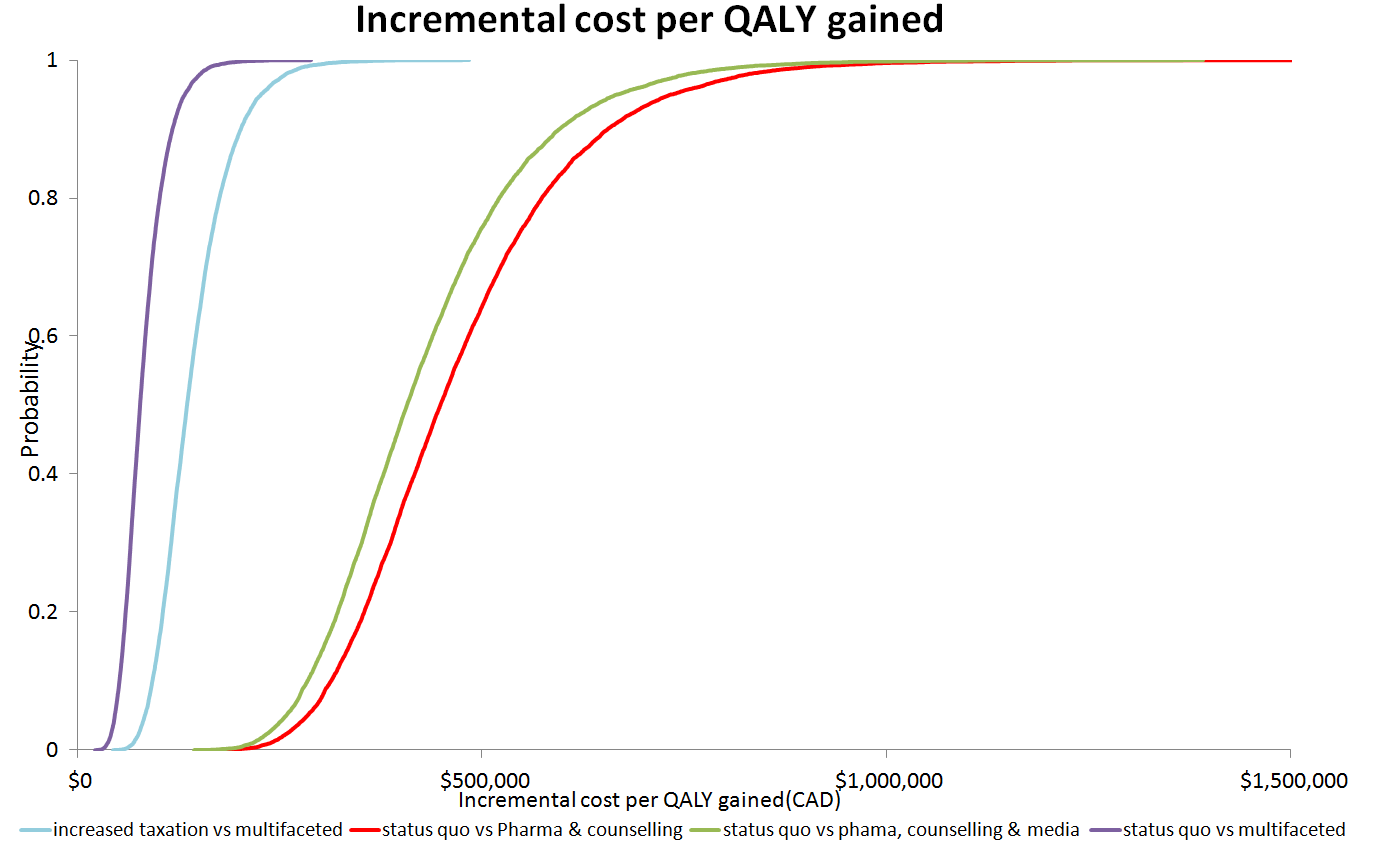


**Table S3. Sensitivity analysis - Projected smoking prevalence over 20 years with different scenarios**

| category | Smokers |
| --- | --- |
| Baseline (2017) | 60% |
| Smoking prevalence after 20 years |  |
| Status quo | 49% |
| Base case – Combined strategy | 39% |
| Assumed pharmacotherapy effectiveness lasting 5 years instead of 3 years – Combined strategy | 38% |
| Assumed continued pharmacotherapy and mass media interventions for 20 years – Combined strategy | 32% |
| 50% of smokers attempt quitting and use pharmacotherapy services – Combined strategy | 21% |
| 75% of smokers attempt quitting and use pharmacotherapy services – Combined strategy | 10% |

# SLIR model equations

## Non-Smoker Stratum

### TB Health States:

- $S_{NS}=Susceptible, non-smokers$
- ${LS}_{NS}=Latent Slow, non-smokers$
- ${LF}_{NS}=Latent Fast, non-smokers$
- $I_{NS}=Infectious, non-smokers$
- ${RL}_{NS}=Recovered from Latent Disease, non-smokers$
- ${RA}_{NS}=Recovered from Active Disease, non-smokers$
- ${RS}_{NS}=Spontaneously Recovered, non-smokers$
- ${RLF}_{NS}=Reinfected from Latent, non-smokers$

### Difference Equations:

1. $S_{NS}\left( t+dt \right)=S_{NS}(t)- S_{NS}(t)*\left( b\frac{I_{NS}(t)}{N}+b\frac{I_{S}(t)}{N} \right)-S_{NS}(t)*d_{rate}+{(S}_{NS}(t)+LS_{NS}(t)+I_{NS}(t)+RL_{NS}(t)+LF_{NS}(t)+RA_{NS}(t)+RS_{NS}(t)+RLF_{NS}(t))*d_{rate}+ I_{NS}(t)*z_{NS}*\left( 1-cd \right)+I_{NS}(t)*cd*zd_{NS}+S_{S}(t)*transition$
2. $LS_{NS}\left( t+dt \right)= LS_{NS}\left( t \right)-LS_{NS}\left( t \right)*prot_{ltbi}*cd_{latent}-LS_{NS}\left( t \right)*v_{NS}-LS_{NS}\left( t \right)*d_{rate}+LF_{NS}\left( t \right)*t-{LS}_{NS}\left( t \right)*\left( b\frac{I_{NS}\left( t \right)}{N}+b\frac{I_{S}\left( t \right)}{N} \right)+RLF_{NS}\left( t \right)*t+LS_{S}(t)*transition$
3. $LF_{NS}\left( t+dt \right)= LF_{NS}\left( t \right)-LF_{NS}\left( t \right)*t+S_{NS}\left( t \right)*\left( b\frac{I_{NS}\left( t \right)}{N}+b\frac{I_{S}\left( t \right)}{N} \right)-LF_{NS}\left( t \right)*p_{NS}-LF_{NS}\left( t \right)*prot_{ltbi}*cd_{latent}-LF_{NS}\left( t \right)*d_{rate}+LF_{S}(t)*transition$
4. $I_{NS}\left( t+dt \right)=I_{NS}(t)-I_{NS}(t)*z_{NS}*\left( 1-cd \right)+{RS}_{NS}(t)*{vSR}_{NS}-I_{NS}(t)*d+{RA}_{NS}(t)*{vAT}_{NS}-I_{NS}(t)*d_{rate}+{LF}_{NS}(t)*p_{NS}+ {LS}_{NS}(t)*v_{NS}-I_{NS}(t)*{tx}_{rate}*cd*txs+{RLF}_{NS}(t)*p_{NS}*im-I_{NS}(t)*cd*{zd}_{NS}+I_{S}(t)*transition$
5. ${RL}_{NS}\left( t+dt \right)={RL}_{NS}\left( t \right)+LS_{NS}\left( t \right)*prot_{ltbi}*cd_{latent}+LF_{NS}\left( t \right)*prot_{ltbi}*cd_{latent}-RL_{NS}\left( t \right)*d_{rate}-{RL}_{NS}\left( t \right)*\left( b\frac{I_{NS}(t)}{N}+b\frac{I_{S}(t)}{N} \right)+RLF_{NS}(t)*prot_{ltbi}*cd_{latent}+RL_{s}\left( t \right)*transition$
6. ${RA}_{NS}\left( t+dt \right)={RA}_{NS}\left( t \right)-RA_{NS}\left( t \right)*d_{rate}-RA_{NS}\left( t \right)*vAT_{NS}+I_{NS}\left( t \right)*tx_{rate}*cd*txs-{RA}_{NS}\left( t \right)*\left( b\frac{I_{NS}(t)}{N}+b\frac{I_{S}(t)}{N} \right)+RA_{S}\left( t \right)*transition$
7. ${RS}_{NS}\left( t+dt \right)={RS}_{NS}\left( t \right)+I_{NS}\left( t \right)*d-RS_{NS}\left( t \right)*d_{rate}-RS_{NS}\left( t \right)*vSR_{NS}-{RS}_{NS}\left( t \right)*\left( b\frac{I_{NS}(t)}{N}+b\frac{I_{S}(t)}{N} \right)+RS_{s}\left( t \right)*transition$
8. ${RLF}_{NS}\left( t+dt \right)={RLF}_{NS}\left( t \right)+{RA}_{NS}\left( t \right)*\left( b\frac{I_{NS}(t)}{N}+b\frac{I_{S}(t)}{N} \right)-RLF_{NS}*p_{NS}*im+{RS}_{NS}\left( t \right)*\left( b\frac{I_{NS}(t)}{N}+b\frac{I_{S}(t)}{N} \right)+{RL}_{NS}\left( t \right)*\left( b\frac{I_{NS}(t)}{N}+b\frac{I_{S}(t)}{N} \right)+{LS}_{NS}\left( t \right)*\left( b\frac{I_{NS}(t)}{N}+b\frac{I_{S}(t)}{N} \right)-RLF_{NS}\left( t \right)*prot_{ltbi}*cd_{latent}-RLF_{NS}\left( t \right)*d_{rate}-RLF_{NS}\left( t \right)*t+RLF_{S}\left( t \right)*transition$

### Initial Conditions:

$$S_{NS}(0)=18,920.7$$

$${LS}_{NS}(0)=48,146.3$$

$${LF}_{NS}\left( 0 \right)=7,074.37$$

$$I_{NS}\left( 0 \right)=2,370.23$$

$${RL}_{NS}\left( 0 \right)=0$$

$${RA}_{NS}\left( 0 \right)=0$$

$${RS}_{NS}\left( 0 \right)=3,030.1$$

$${RLF}_{NS}\left( 0 \right)=20,258.3$$

## Smoker Stratum

### TB Health States:

- $S_{S}=Susceptible, smokers$
- ${LS}_{S}=Latent Slow, smokers$
- ${LF}_{S}=Latent Fast, smokers$
- $I_{S}=Infectious, smokers$
- ${RL}_{S}=Recovered from Latent Disease, smokers$
- ${RA}_{S}=Recovered from Active Disease, smokers$
- ${RS}_{S}=Spontaneously Recovered, smokers$
- ${RLF}_{S}=Reinfected from Latent, smokers$

### Difference Equations:

1. $S_{S}\left( t+dt \right)=S_{S}\left( t \right)- S_{S}\left( t \right)*\left( b\frac{I_{NS}\left( t \right)}{N}+b\frac{I_{S}\left( t \right)}{N} \right)*1.9-S_{S}(t)*d_{rate}+{(S}_{S}(t)+LS_{S}(t)+I_{S}(t)+RL_{S}(t)+LF_{S}(t)+RA_{S}(t)+RS_{S}(t)+RLF_{S}(t))*d_{rate}+ I_{S}(t)*z_{S}*\left( 1-cd \right)+I_{S}(t)*cd*zd_{S}-S_{S}(t)*transition$
2. $LS_{S}\left( t+dt \right)= LS_{S}\left( t \right)-LS_{S}\left( t \right)*prot_{ltbi}*cd_{latent}-LS_{S}\left( t \right)*v_{S}-LS_{S}\left( t \right)*d_{rate}+LF_{S}\left( t \right)*t-{LS}_{S}\left( t \right)*\left( b\frac{I_{NS}\left( t \right)}{N}+b\frac{I_{S}\left( t \right)}{N} \right)*1.9+RLF_{S}\left( t \right)*t-LS_{S}(t)*transition$
3. $LF_{S}\left( t+dt \right)= LF_{S}\left( t \right)-LF_{S}\left( t \right)*t+S_{S}\left( t \right)*\left( b\frac{I_{NS}\left( t \right)}{N}+b\frac{I_{S}\left( t \right)}{N} \right)*1.9-LF_{S}\left( t \right)*p_{S}-LF_{S}\left( t \right)*prot_{ltbi}*cd_{latent}-LF_{S}\left( t \right)*d_{rate}-LF_{S}(t)*transition$
4. $I_{S}\left( t+dt \right)=I_{S}\left( t \right)-I_{S}\left( t \right)*z_{S}*\left( 1-cd \right)+{RS}_{S}\left( t \right)*{vSR}_{S}-I_{S}\left( t \right)*d+{RA}_{S}\left( t \right)*{vAT}_{S}-I_{S}\left( t \right)*d_{rate}+{LF}_{S}\left( t \right)*p_{S}+ {LS}_{S}\left( t \right)*v_{S}-I_{S}\left( t \right)*{tx}_{rate}*cd*txs+{RLF}_{S}\left( t \right)*p_{S}*im-I_{S}\left( t \right)*cd*{zd}_{S}-I_{S}(t)*transition$
5. ${RL}_{S}\left( t+dt \right)={RL}_{S}\left( t \right)+LS_{S}\left( t \right)*prot_{ltbi}*cd_{latent}+LF_{S}\left( t \right)*prot_{ltbi}*cd_{latent}-RL_{S}\left( t \right)*d_{rate}-{RL}_{S}\left( t \right)*\left( b\frac{I_{NS}\left( t \right)}{N}+b\frac{I_{S}\left( t \right)}{N} \right)*1.9+RLF_{S}\left( t \right)*prot_{ltbi}*cd_{latent}-RL_{s}\left( t \right)*transition$
6. ${RA}_{S}\left( t+dt \right)={RA}_{S}\left( t \right)-RA_{S}\left( t \right)*d_{rate}-RA_{S}\left( t \right)*vAT_{S}+I_{S}\left( t \right)*tx_{rate}*cd*txs-{RA}_{S}\left( t \right)*\left( b\frac{I_{NS}\left( t \right)}{N}+b\frac{I_{S}\left( t \right)}{N} \right)*1.9-RA_{S}\left( t \right)*transition$
7. ${RS}_{S}\left( t+dt \right)={RS}_{S}\left( t \right)+I_{S}\left( t \right)*d-RS_{S}\left( t \right)*d_{rate}-RS_{S}\left( t \right)*vSR_{S}-{RS}_{S}\left( t \right)*\left( b\frac{I_{NS}\left( t \right)}{N}+b\frac{I_{S}\left( t \right)}{N} \right)*1.9-RS_{s}\left( t \right)*transition$
8. ${RLF}_{S}\left( t+dt \right)={RLF}_{S}\left( t \right)+{RA}_{S}\left( t \right)*\left( b\frac{I_{NS}\left( t \right)}{N}+b\frac{I_{S}\left( t \right)}{N} \right)*1.9-RLF_{S}*p_{S}*im+{RS}_{S}\left( t \right)*\left( b\frac{I_{NS}\left( t \right)}{N}+b\frac{I_{S}\left( t \right)}{N} \right)*1.9+{RL}_{S}\left( t \right)*\left( b\frac{I_{NS}\left( t \right)}{N}+b\frac{I_{S}\left( t \right)}{N} \right)*1.9+{LS}_{S}\left( t \right)*\left( b\frac{I_{NS}\left( t \right)}{N}+b\frac{I_{S}\left( t \right)}{N} \right)*1.9-RLF_{S}\left( t \right)*prot_{ltbi}*cd_{latent}-RLF_{S}\left( t \right)*d_{rate}-RLF_{S}\left( t \right)*t-RLF_{S}\left( t \right)*transition$

### Initial Conditions:

$$S_{S}(0)=128,372$$

$${LS}_{S}(0)=362,487$$

$${LF}_{S}(0)=85,079.6$$

$$I_{S}\left( 0 \right)=26,235.1$$

$${RL}_{S}\left( 0 \right)=0$$

$${RA}_{S}\left( 0 \right)=0$$

$${RS}_{S}\left( 0 \right)=19,474.9$$

$${RLF}_{S}\left( 0 \right)=278,351$$

## Parameter Values

### Pathogenetic Parameters (Non-Smokers):

$$p_{NS}=if t<52 then 0.055e^{-0.0075t} else 0.0372+0.00045(t-52)$$

$$v_{NS}=if t \leq52 then 0.001-0.0000096t else 0.0005$$

$$z_{NS}=0.2$$

$${zd}_{NS}=0.042$$

$${vSR}_{NS}=0.025$$

$${vAT}_{NS}=0.015$$

### Pathogenetic Parameters (Smokers):

$$p_{S}=p_{NS}*1.5$$

$$v_{S}=v_{NS}*1.5$$

$$z_{S}=z_{NS}*2.6$$

$${zd}_{S}=zd_{NS}*2.6$$

$${vSR}_{S}=vSR_{NS}*1.5$$

$${vAT}_{S}=vAT_{NS}*1.5$$

### Other Parameters (Common to Non-Smokers & Smokers):

$$t=0.3$$

$$d=0.25$$

$$im=0.55$$

$$cd_{latent}=if t<20 then 0 else if 20\leq t<45 then 0.0012\left( t-20 \right) else 0.03$$

$$cd= if t<20 then 0 else if 20\leq t<30 then 0.08\left( t-20 \right)+0.1 else 0.9$$

$$tx_{rate}=1$$

$$txs=0.97$$

$$prot_{ltbi}=0.4833$$

$$N=1000000 \left\{ total population \right\}$$

$$d_{rate}=0.000005t^{2}-0.0004t+0.023$$

$$b=if t<40 then 5e^{-0.025t} else if 40\leq t<52 then 1.84 else 1.84e^{0.04\left( t-52 \right)}$$

$transition=if t<20 then 0 else if 20\leq t<68 then-0.000024t^{2}+0.002106t-0.033237 else 0 \{transition from smokers to non-smokers\}$

# References

1. Grzybowski S, Enarson DA. The fate of cases of pulmonary tuberculosis under various treatment programmes. Bull IUAT. 1978;53(2):70-5

2. Barsh RL. Canada’s Aboriginal peoples: Social integration or disintegration. The Canadian Journal of Native Studies. 1994;14(1):1-46

3. Grzybowski S, Dorken E. Tuberculosis in Inuit. Ecol Dis. 1983;2(2):145-8

4. Grzybowski S, Styblo K, Dorken E. Tuberculosis in eskimos. Tubercle. 1976;57(4):S1-S58

5. MacDonald N, Hébert PC, Stanbrook MB. Tuberculosis in Nunavut: a century of failure. CMAJ. 2011

6. Lee RS, Proulx J-F, Menzies D, Behr MA. Progression to tuberculosis disease increases with multiple exposures. Eur Respir J. 2016;10.1183/13993003.00893-2016.<http://erj.ersjournals.com/content/48/6/1682>

7. Beaudry PH. Pulmonary function survey of the Canadian Eastern Arctic Eskimo. Arch Environ Health. 1968;17(4):524-8

8. Carrière G, Tjepkema M, Pennock J, Goedhuis N. Cancer patterns in Inuit Nunangat: 1998–2007. Int J Circumpolar Health. 2012;71(1):18581.<http://www.tandfonline.com/doi/pdf/10.3402/ijch.v71i0.18581?needAccess=true>

9. Millar WJ. Place of birth and ethnic status: factors associated with smoking prevalence among Canadians. Health Rep. 1992;4(1):7-24

10. Millar WJ. Smoking prevalence in the Canadian Arctic. Arctic Med Res. 1990;49:23-8

11. Pickering J, Lavallee C, Hanley J. Cigarette smoking in Cree Indian school children of the James Bay region. Arctic Med Res. 1989;48(1):6-11

12. Rode A, Shephard R. Lung function in Canadian Inuit: a follow-up study. Can Med Assoc J. 1984;131(7):741.<https://www.ncbi.nlm.nih.gov/pmc/articles/PMC1483593/pdf/canmedaj00369-0039.pdf>

13. Tait H. Aboriginal Peoples Survey, 2006: Inuit health and social conditions: Statistics Canada, Social and Aboriginal Statistics Division Ottawa; 2008.

14. Statistics Canada. Smokers, by sex, provinces and territories (Percent). 2016.

15. Statistics Canada. Canadian health characteristics, two year period estimates, by age group and sex, Canada, provinces, territories and health regions. 2017.

16. Interval C. Smoking, 2016 - Statistics Canada. Statistics Canada. 2017;2016(82-625-X).<https://www.statcan.gc.ca/pub/82-625-x/2017001/article/54864-eng.htm>

17. Canadian Human Mortality Database. Canada - Northwest Territories & Nunavut, Life expectancy at birth (period, 1x1). In: Demography Do, editor. Université de Montréal (Canada)2018.[www.demo.umontreal.ca/chmd/](file:///C:\Users\Olivia.Oxlade\Desktop\KS%20projects\Dynamic%20smoking%20model\BMC%20MEd%20Submission\www.demo.umontreal.ca\chmd\)

18. Nolan CM, Elarth AM. Tuberculosis in a cohort of Southeast Asian refugees. Am Rev Respir Dis. 1988;137:805-9

19. Comstock GW, Edwards LB, Livesay VT. Tuberculosis Morbidity in the US Navy: Its Distribution and Decline 1, 2. Am Rev Respir Dis. 1974;110(5):572-80

20. Behr MA, Edelstein PH, Ramakrishnan L. Revisiting the timetable of tuberculosis. BMJ. 2018;362:k2738.<https://www.bmj.com/content/bmj/362/bmj.k2738.full.pdf>

21. Grzybowski S. Drugs are not enough: failure of short-course chemotherapy in a district in India. Tuber Lung Dis. 1993;74(3):145-6

22. Tiemersma EW, van der Werf MJ, Borgdorff MW, Williams BG, Nagelkerke NJ. Natural history of tuberculosis: duration and fatality of untreated pulmonary tuberculosis in HIV negative patients: a systematic review. PloS one. 2011;6(4):e17601.<https://www.ncbi.nlm.nih.gov/pmc/articles/PMC3070694/pdf/pone.0017601.pdf>

23. Sutherland I, Švandová E, Radhakrishna S. The development of clinical tuberculosis following infection with tubercle bacilli: 1. A theoretical model for the development of clinical tuberculosis following infection, linking from data on the risk of tuberculous infection and the incidence of clinical tuberculosis in the Netherlands. Tubercle. 1982;63(4):255-68

24. Andrews JR, Noubary F, Walensky RP, Cerda R, Losina E, Horsburgh CR. Risk of Progression to Active Tuberculosis Following Reinfection With Mycobacterium tuberculosis. Clin Infect Dis. 2012;54(6):784-91.<http://dx.doi.org/10.1093/cid/cir951>

25. NUNAVUT DEPARTMENT OF HEALTH. Nunavut TB Manual 2017: NUNAVUT DEPARTMENT OF HEALTH; 2017. <https://www.gov.nu.ca/sites/default/files/nunavut_tb_manual_2017.pdf>.

26. Centre for Communicable Diseases and Infection Control. Canadian Tuberculosis Standards. Public Health Agency, Canada. 2014;7th edition.[www.phac-aspc.gc.ca](file:///C:\Users\Olivia.Oxlade\Desktop\KS%20projects\Dynamic%20smoking%20model\BMC%20MEd%20Submission\www.phac-aspc.gc.ca)

27. Alvarez GG, VanDyk DD, Aaron SD, et al. Taima (stop) TB: the impact of a multifaceted TB awareness and door-to-door campaign in residential areas of high risk for TB in Iqaluit, Nunavut. PloS one. 2014;9(7):e100975

28. Canadian Tobacco Alcohol and Drugs (CTADS). Canadian Tobacco Alcohol and Drugs (CTADS): 2015 summary. 2015;Summary of results for 2015.<https://www.canada.ca/en/health-canada/services/canadian-tobacco-alcohol-drugs-survey/2015-summary.html>

29. White CM, Rynard VL, Reid JL, Ahmed R, Burkhalter R, Hammond D. Stop-Smoking Medication Use, Subsidization Policies, and Cessation in Canada. Am J Prev Med. 2015;49(2):188-98.<http://www.ajpmonline.org/article/S0749-3797(15)00091-4/fulltext>

30. Prochaska JJ, Delucchi K, Hall SM. A meta-analysis of smoking cessation interventions with individuals in substance abuse treatment or recovery. J Consult Clin Psychol. 2004;72(6):1144.<https://cloudfront.escholarship.org/dist/prd/content/qt0r8673wv/qt0r8673wv.pdf?t=lnrsdv>

31. Stead LF, Perera R, Bullen C, et al. Nicotine replacement therapy for smoking cessation. Cochrane Database Syst Rev. 2012;10.1002/14651858.CD000146.pub4(11).<http://dx.doi.org/10.1002/14651858.CD000146.pub4>

32. Stead LF, Koilpillai P, Lancaster T. Additional behavioural support as an adjunct to pharmacotherapy for smoking cessation. Cochrane Database Syst Rev. 2015;10.1002/14651858.CD009670.pub3(10).<http://dx.doi.org/10.1002/14651858.CD009670.pub3>

33. Levy D, de Almeida LM, Szklo A. The Brazil SimSmoke policy simulation model: the effect of strong tobacco control policies on smoking prevalence and smoking-attributable deaths in a middle income nation. PLoS Med. 2012;9(11):e1001336.<https://www.ncbi.nlm.nih.gov/pmc/articles/PMC3491001/pdf/pmed.1001336.pdf>

34. Levy DT, Chaloupka F, Gitchell J. The effects of tobacco control policies on smoking rates: a tobacco control scorecard. J Public Health Manag Pract. 2004;10(4):338-53.<http://ovidsp.tx.ovid.com/ovftpdfs/FPDDNCGCFCDFCG00/fs047/ovft/live/gv031/00124784/00124784-200407000-00011.pdf>

35. Marley JV, Atkinson D, Kitaura T, et al. The Be Our Ally Beat Smoking (BOABS) study, a randomised controlled trial of an intensive smoking cessation intervention in a remote aboriginal Australian health care setting. BMC public health. 2014;14(1):32

36. Gallet CA, List JA. Cigarette demand: a meta‐analysis of elasticities. Health Econ. 2002;12(10):821-35.<http://dx.doi.org/10.1002/hec.765>

37. Levy DT, Cummings KM, Hyland A. Increasing taxes as a strategy to reduce cigarette use and deaths: results of a simulation model. Prev Med. 2000;31(3):279-86

38. Nunavut Department of Finance. Tobacco tax increase. News release March 15, 2017. <https://www.gov.nu.ca/finance/news/tobacco-tax-increase>.
